# RDE-DR: robust deep ensemble CNNs for automated diabetic retinopathy detection from fundus images

**DOI:** 10.1038/s41598-026-48669-y

**Published:** 2026-05-17

**Authors:** Ishaq Aiche, Youcef Brik, Bilal Attallah, Oussama Bouguerra, Abdelaziz Rabehi, Mustapha Habib, Doaa Sami Khafaga, El-Sayed M. El-kenawy

**Affiliations:** 1 Laboratory of Signal and Systems Analysis (LASS), Department of Electronics, Faculty of Technology, University of M’sila, Ichebilia, PO Box 166, M’sila, 28000 Algeria; 2 Laboratory of Telecommunication and Smart Systems (LTSS), Faculty of Science and Technology, University of Djelfa, PO Box 3117, Djelfa, 17000 Algeria; 3 Civil and Architectural Engineering, KTH Royal Institute of Technology, Teknikringen, 78, 11428 Stockholm, Sweden; 4 Department of Computer Sciences, College of Computer and Information Sciences, Princess Nourah Bint Abdulrahman University, Riyadh, 11671 Saudi Arabia; 5 Department for Communications and Electronics, Delta Higher Institute of Engineering and Technology, Mansoura, 35511 Egypt; 6 Jadara Research Center, Jadara University, Irbid , 21110 Jordan; 7 Laboratory of Electrical Engineering (LGE), Faculty of Technology, University of M’sila, PO Box 166 Ichebilia, 28000 M’sila, Algeria

**Keywords:** Diabetic retinopathy, Fundus images, Transfer learning, Image preprocessing, Deep ensemble learning, Threshold optimization, Computational biology and bioinformatics, Diseases, Health care, Mathematics and computing, Medical research

## Abstract

Diabetic retinopathy (DR) is a leading cause of preventable blindness, motivating the development of reliable automated screening systems. This work proposes a Robust Deep Ensemble for Diabetic Retinopathy detection (RDE-DR) by analyzing ensemble fusion strategies. Four pre-trained convolutional neural networks (ResNet50, VGG16, VGG19, and DenseNet121) are trained using CLAHE-enhanced APTOS 2019 fundus images and integrated through seven heterogeneous fusion mechanisms, including voting-based, rank-based, and fuzzy-integral-inspired strategies. A consistent evaluation protocol is adopted, incorporating threshold optimization and probabilistic calibration analysis to validate robustness, decision margins, and accuracy–precision trade-offs. Experimental results show that multiple fusion techniques achieve comparable high performance and stable behavior on the APTOS 2019 benchmark, with the best configuration reaching 98.64% accuracy, 98.40% precision, 98.92% recall, 98.66% F1-score, and 99.78% Area-Under-Curve (AUC). Beyond peak accuracy, the study provides insights into ensemble reliability, calibration characteristics, and practical design choices for medical image classification systems. These results show that integrating transfer learning with CLAHE preprocessing and ensemble fusion yields stable experimental performance on the APTOS 2019 benchmark, suggesting potential for future medical decision support.

## Introduction

Diabetic retinopathy (DR) is a leading cause of vision impairment and preventable blindness worldwide, particularly among individuals with diabetes^1^. Early and accurate screening is essential to prevent irreversible vision loss and improve patient outcomes^2^. However, early-stage DR detection remains challenging due to the subtle appearance of pathological features and the reliance on manual interpretation by clinical experts, which is time-consuming and subject to inter-observer variability^3^. In recent years, advances in artificial intelligence (AI) and deep learning have significantly enhanced automated disease screening, enabling reliable analysis of retinal fundus images^4^. Convolutional neural networks (CNNs), in particular, have demonstrated strong performance in medical image classification and segmentation tasks. Nevertheless, the scarcity of large, well-annotated medical datasets limits the training of high-capacity models from scratch^5^. Transfer learning alleviates this limitation by adapting models pre-trained on large-scale datasets, such as ImageNet, including ResNet50, VGG16, VGG19, and DenseNet121, to domain-specific tasks such as DR detection^6^.

Building upon transfer learning, ensemble learning has emerged as an effective strategy for improving robustness, generalization, and reliability in medical image analysis^7^. Instead of relying on a single model, ensembles integrate complementary predictions from multiple architectures to reduce variance and mitigate individual model bias^8^. Many works report performance improvements using voting or averaging techniques but do not analyze how fusion design influences calibration reliability, decision margins, or operational stability under identical experimental conditions. In addition, fixed decision thresholds are commonly adopted without investigating their impact on accuracy–precision trade-offs, which is critical for medical screening scenarios. Furthermore, comparisons across studies are frequently hindered by inconsistent preprocessing pipelines, training protocols, and evaluation settings, making it difficult to draw reproducible conclusions regarding ensemble effectiveness.

To address these limitations, this work proposes a Robust Deep Ensemble for Diabetic Retinopathy Detection (RDE-DR). This unified and reproducible experimental framework integrates four pretrained CNN backbones (ResNet50, VGG16, VGG19, and DenseNet121) into an automated DR screening system. To systematically exploit model complementarity, seven fusion strategies are investigated, such as hard voting, soft voting, weighted soft voting, rank-based fusion, Choquet integral, Sugeno integral, and average-logit fusion^9,10^. These fusion strategies are involved under controlled conditions, probabilistic behavior analysis and threshold considerations. The experiments are conducted on the APTOS 2019 Blindness Detection dataset, incorporating contrast-limited adaptive histogram equalization (CLAHE) and data augmentation techniques^11^.

Rather than pursuing incremental performance gains, the study emphasizes methodological understanding and practical guidance for designing reliable ensemble-based medical image classifiers. The main contributions of this work are as follows:

(1) A unified experimental pipeline integrating CLAHE-based preprocessing, transfer learning with four heterogeneous CNN architectures (ResNet50, VGG16, VGG19, DenseNet121), and seven different ensemble fusion strategies within identical training and evaluation conditions.

(2) A comprehensive comparative analysis of voting-based, rank-based, and fuzzy-integral-based fusion mechanisms, enabling controlled assessment of their robustness, calibration behavior, and metric stability.

(3) Systematic threshold optimization applied consistently across all fusion strategies to study accuracy–precision trade-offs and operational flexibility in medical screening scenarios.

(4) Probabilistic behavior analysis using Kernel Density Estimation (KDE) to characterize decision margins and reliability beyond conventional accuracy reporting.

The remainder of this paper is organized as follows: Sect.  2 reviews related work on DR detection using deep and ensemble learning. Section  3 presents the methodology, including CLAHE preprocessing, model architectures, and fusion strategies. Section  4 describes the experimental setup, evaluation metrics, and results. Section  5 compares the proposed framework performance with similar works. Finally, Sect.  6 concludes the paper and outlines future research directions.

## Related work

Several studies using powerful pre-trained convolutional neural networks (CNNs), such as ResNet50, VGG16/19, DenseNet121, and EfficientNet, have reported DR screening accuracies typically in the range of 94–97%, with area under the ROC curve (AUC) values of around 0.97–0.99 on public datasets such as APTOS 2019 and other Kaggle DR challenges. These studies have also reported sensitivities and specificities often exceeding 94%^12^. Early approaches combined machine learning with deep features, demonstrating that ensembles of CNN-derived features fed to classic classifier can outperform standalone models^13^.

Integrating multiple CNN architectures via ensemble or model-fusion approaches is another significant development in the field^14^. It has been proven that combining the predictive strengths of different models using some powerful fusion techniques consistently produces better results than single-model baselines. For example, an ensemble framework proposed in^15^ achieved an accuracy of around 96–97%, F1-scores above 0.96, and an AUC greater than 0.98 for binary DR classification, outperforming its best individual CNN by 2–3% points in terms of both accuracy and sensitivity. Similarly, an ensemble-based DR system using optimized voting and feature-level fusion presented in^16^ achieved around 95–96% accuracy and close to 95% sensitivity and specificity, as well as AUC values above 0.97 on benchmark datasets.

Islam et al.^17^ presented deep ensembles integrating multiple CNNs via feature-level fusion and probability voting/stacking. The authors evaluated pairwise and tri-fusion of pretrained CNN backbones (ResNet50, EfficientNet-B0, DenseNet121) for binary DR screening. Their results indicate that fused feature representations consistently outperform individual models, while offering promising computational efficiency trade-offs.

Moreover, Lin^18^ proposed an accuracy-weighted ensemble framework that combines seven distinct CNN architectures (including ResNet-50, DenseNet variants, EfficientNet, and MobileNet models) using a weighted majority voting scheme coupled with entropy-guided uncertainty estimation. This approach not only improved classification performance (achieving near 99% accuracy post-filtering) but also enabled rejection of low-confidence predictions.

Similarly, soft voting ensembles have been proposed in^19^ where posterior class probabilities from several pretrained networks (EfficientNet-B0, ResNet-50, and DenseNet-121) are aggregated to enhance the final decision. The obtained results reported noticeable gains in detection accuracy relative to individual CNN baselines, validating the utility of probabilistic fusion mechanisms in fundus image classification.

More recently, many studies in medical image analysis have increasingly explored advanced architectural paradigms beyond conventional CNNs, including transformer-based backbones, attention mechanisms, hybrid CNN–Transformer architectures, explainable learning frameworks, and secure collaborative infrastructures^20,21^. Hybrid models combining convolutional networks with Vision Transformers and multi-scale fusion have demonstrated improved spatial representation and segmentation accuracy in medical image analysis (brain tumor, chest, cardiomegaly, and fundus images) highlighting the effectiveness of global–local feature integration^22–25^. Likewise, attention-enhanced architectures such as DeepLabV3 with attention modules and EfficientNet-based explainable frameworks incorporating Grad-CAM have shown improved localization accuracy and interpretability on both public and clinical datasets^26^.

In parallel, image pre-processing techniques such as Contrast-Limited Adaptive Histogram Equalization (CLAHE) are often used to improve the visibility of vessels and lesions. This can reduce illumination variability and enhance the robustness of downstream models. Kobat et al.^27^ reported an increase from approximately 93% to 96% and from 0.95 to 0.98 in term of accuracy and AUC, respectively, after applying CLAHE and related enhancement steps. To facilitate the recap of previous studies, Table 1 summarizes the most important works on automated DR detection, highlighting the datasets used, number of images, learning architectures, methodological strategies, and reported performance metrics.

**Table 1:** Overview of representative studies on automated diabetic retinopathy detection.

Ref.	Year	Dataset	No. of images	Task	Model/architecture	Methodology	Results
^12^	2018	Kaggle EyePACS	~ 35,000	Multi-class	InceptionV3	Transfer learning with augmentation	Acc ≈ 93%
^13^	2025	Kaggle DR datasets (including APTOS-type data)	~ 30,000 (reported across Kaggle datasets)	Binary	VGG16, EfficientNetB0	Gaussian filtering + transfer learning	Accuracy ≈ 97.54%
^14^	2019	Messidor-2	1748	Binary	VGG16	Fine-tuning + preprocessing	Acc ≈ 95%
^17^	2025	APTOS 2019 (primary)	3662	Binary	CNN fusion models (EfficientNet variants + lightweight CNNs)	Transfer learning + feature-level fusion + optimized ensemble strategy	Accuracy ≈ 97–98%, AUC > 0.97 (reported), improved efficiency–accuracy trade-off
^18^	2025	EyePACS (primary), APTOS (reported)	~ 35,000 (EyePACS), 3662 (APTOS)	Binary (referable vs. non-referable DR)	Accuracy-weighted deep ensemble (CNN backbones)	Transfer learning + weighted ensemble + entropy-guided abstention (selective prediction)	Accuracy ≈ 96–98%, AUC > 0.97; improved reliability through abstention mechanism
^19^	2024	APTOS 2019	3662	Binary	Deep CNN ensemble (VGG, ResNet variants)	Transfer learning + soft voting ensemble	Accuracy ≈ 96–97%, AUC reported (> 0.97)
^20^	2021	EyePACS	~ 80,000	Multi-class	DenseNet121	Deep CNN with augmentation	AUC ≈ 0.97
^21^	2022	APTOS + Messidor	~ 5000	Binary	MobileNetV2	Lightweight CNN	Acc ≈ 94%
^22^	2023	APTOS 2019	3662	Binary	Ensemble (VGG + ResNet)	Majority voting	Acc ≈ 97%
^27^	2020	APTOS 2019	3662	Binary	ResNet50	CLAHE + transfer learning	Acc ≈ 96%

Despite the aforementioned advancements, several gaps remain in the literature that motivate the development of more rigorous ensemble fusion frameworks based explicitly on pretrained CNNs. Many existing ensembles rely on empirical combinations of a small number of architectures or simple voting schemes, without systematically exploring feature- and decision-level fusion strategies tailored to DR lesion patterns. Besides, there is still limited work on unified frameworks that jointly optimize transfer learning from multiple pretrained CNNs, fusion mechanisms, and calibration of predicted probabilities for clinical deployment.

The present work builds upon the aforementioned trends by designing an ensemble fusion framework that integrates several pretrained CNN models specialized for retinal imaging, combining their outputs through a carefully designed fusion strategy for robust DR screening. By leveraging the complementary strengths of different pretrained models and explicitly addressing probabilistic prediction behavior and threshold considerations, the proposed framework aims to improve upon current ensemble approaches and contribute a more reliable tool for large-scale DR screening systems.

## Materials and methods

### Proposed method

**Fig. 1:**
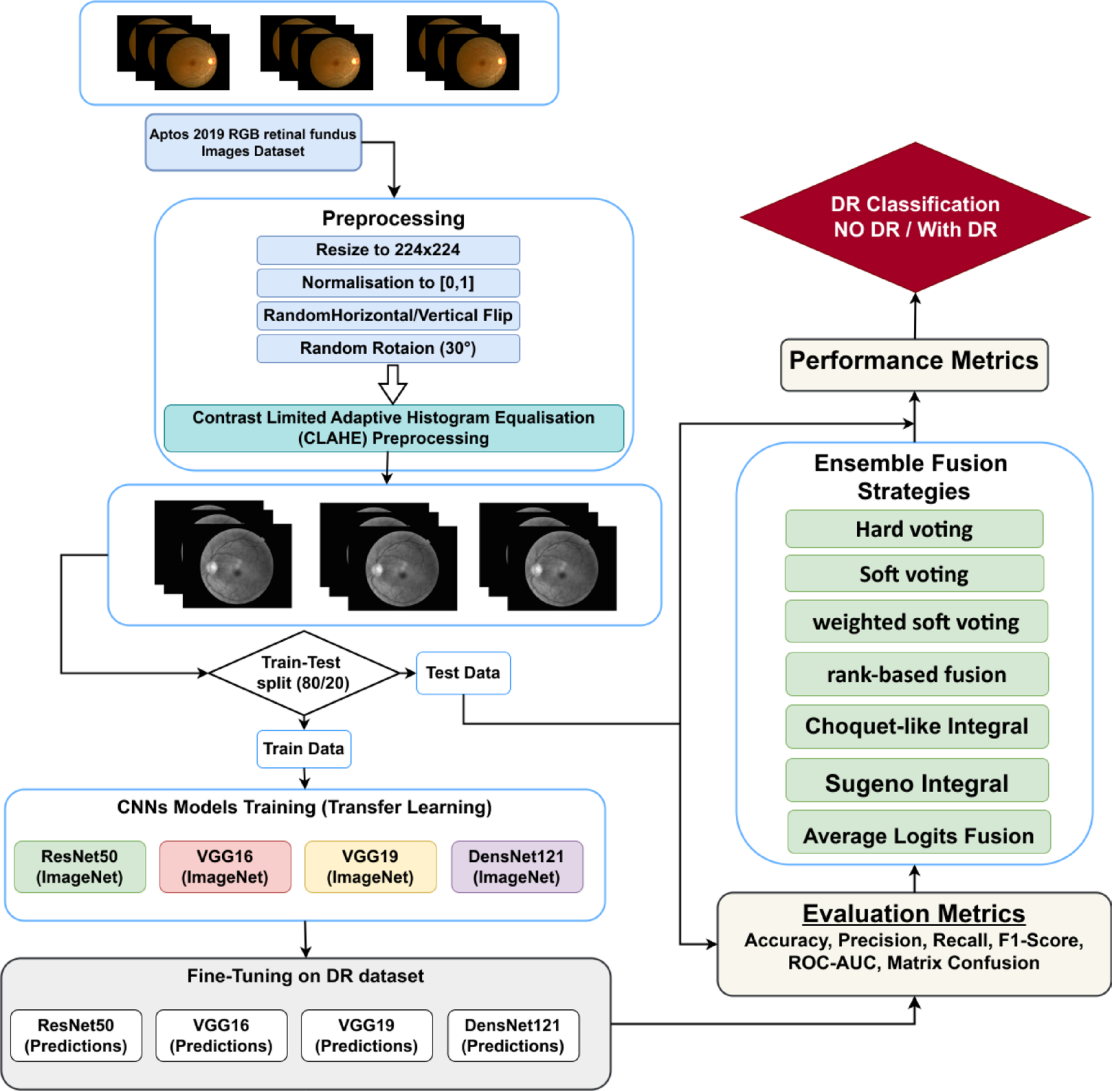
Schematic representation of the RDE‑DR framework for automated diabetic retinopathy detection using CLAHE‑enhanced APTOS 2019 fundus images and multi‑strategy ensemble fusion.

Our study proposes a deep ensemble transfer learning framework (RDE-DR) for the automated detection of diabetic retinopathy (DR) using CLAHE-enhanced APTOS 2019 fundus images. The pipeline begins with the APTOS 2019 RGB retinal fundus dataset^28^, wher DR images are resized, normalized, and augmented to improve generalization^29^. CLAHE is then applied to enhance local contrast and highlight diagnostically relevant structures, including microaneurysms, exudates, and hemorrhages^30^. The pre-processed images are split into training and testing sets (80/20), and four pre-trained convolutional neural networks, ResNet50, VGG16, VGG19, and DenseNet121, are trained via transfer learning from ImageNet^31^ weights for binary DR classification (No_DR and DR). Seven ensemble fusion strategies are employed to exploit the complementary representations learned by these architectures: hard voting, soft voting, weighted soft voting, rank-based fusion, Choquet-like integral, Sugeno integral, and average logits fusion. These strategies produce a robust aggregated prediction for each image. The overall system is evaluated using accuracy, precision, recall, the F1 score, the ROC-AUC, and confusion matrices. The full RDE-DR pipeline is summarized schematically in Fig. 1.

### Dataset

This study uses the Asia Pacific Tele-Ophthalmology Society (APTOS) 2019 Blindness Detection Dataset only^28^, focusing on the labels for diabetic retinopathy (DR) provided. Images labeled with any degree of DR (mild, moderate, severe, or proliferative) are grouped in class 1 (DR), while images without DR are assigned to class 0 (No_DR). This binary reformulation simplifies the screening task, making it a matter of distinguishing between diseased and healthy retinas, which is consistent with many automated pre-screening scenarios.

The APTOS 2019 dataset was selected because it provides a thorough representation of retinal abnormalities at various DR severity levels and is widely used in research settings to benchmark computer-aided diagnosis systems. The original dataset comprises 3662 retinal fundus images with ground-truth labels, and an additional 1928 images form a separate test set without public labels. This study only uses the 3662 labeled training images, of which 1805 belong to the No_DR class (class 0) and 1857 to the DR class (class 1). Focusing on a single, high-quality dataset ensures consistent preprocessing, training, and evaluation protocols throughout the study.

**Fig. 2:**
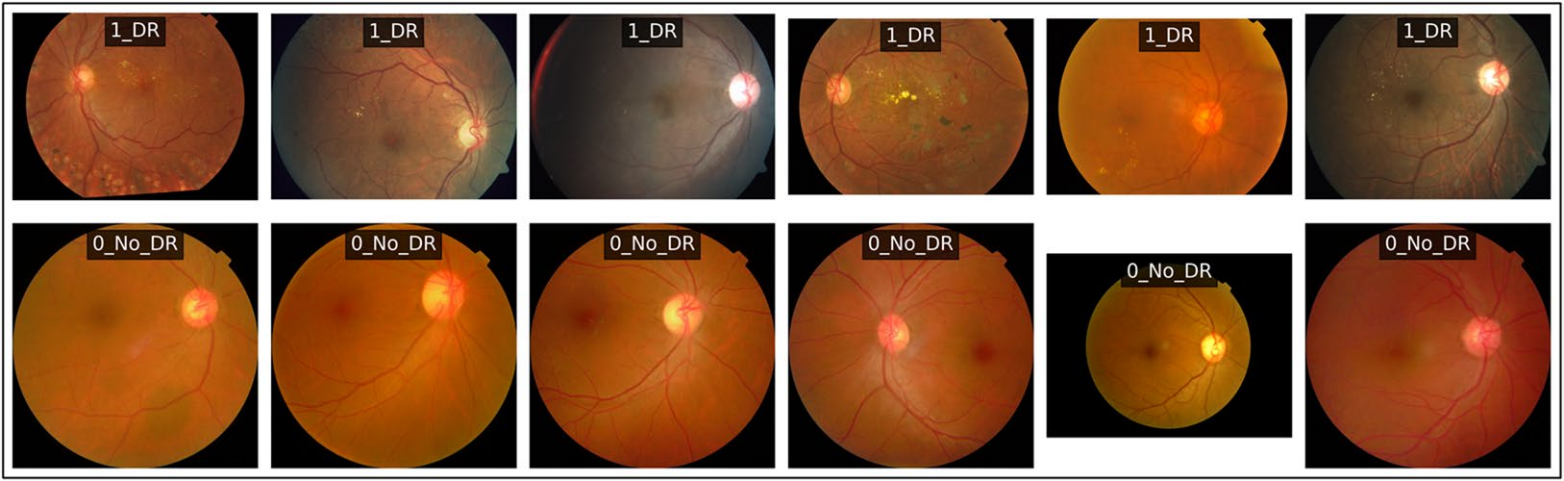
Representative fundus images from the APTOS 2019 study for the two classes: diabetic retinopathy (DR) and no-diabetic (No_DR).

We recall here that APTOS 2019 dataset does not provide explicit patient identifiers or paired left-right eye metadata. Therefore, it was not possible to perform a strict patient-level split. Figure 2 shows examples of images from both classes, demonstrating the variety of appearances, lighting conditions, and pathologies within the APTOS 2019 dataset.

### Data pre-processing

**Fig. 3:**
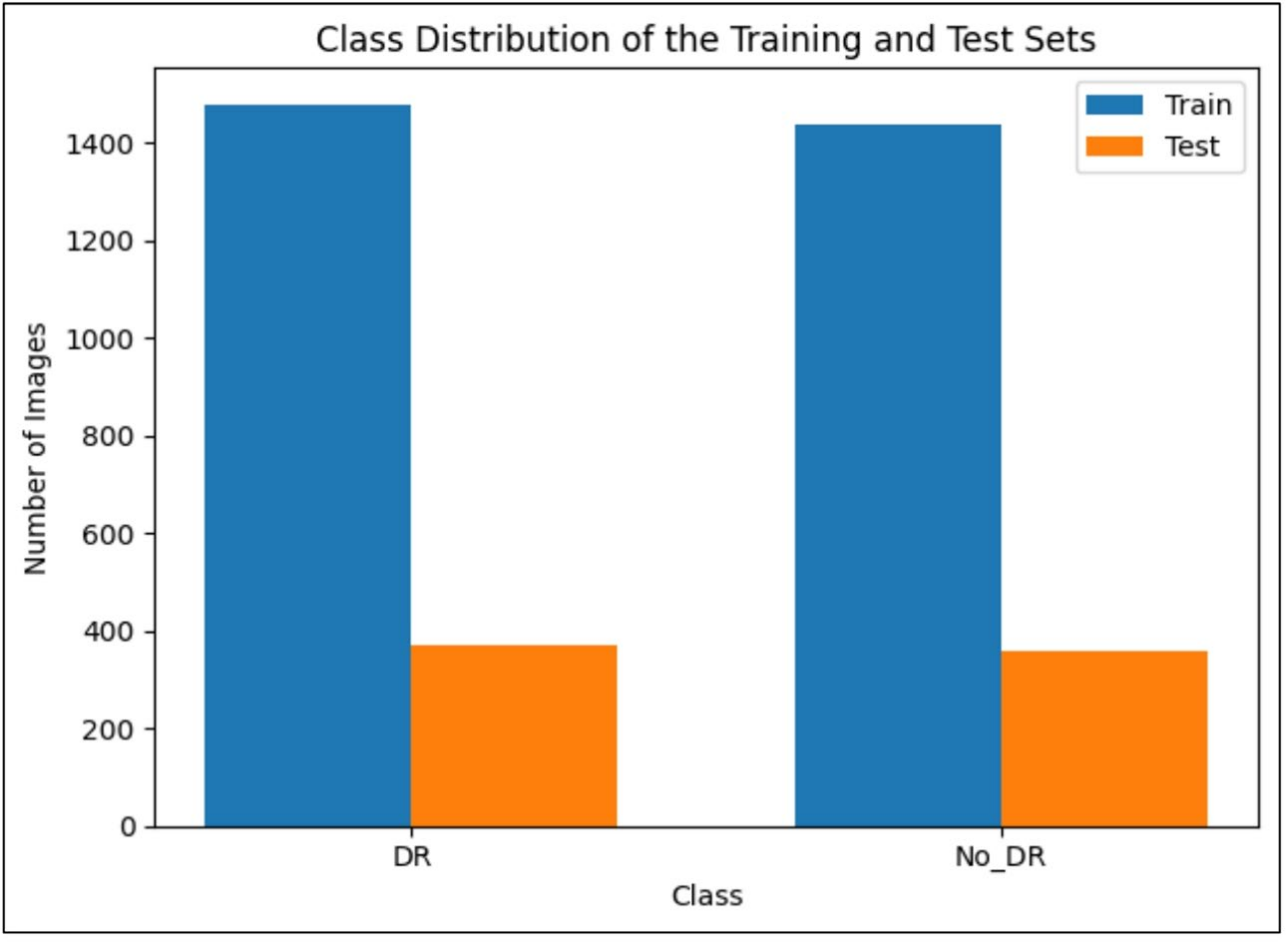
Distribution of DR and No_DR images in the training and test subsets of the APTOS 2019 dataset.

This section outlines the preparation steps applied to the APTOS 2019 images prior to training the model. The original retinal fundus images are high-resolution (typically around 4288 × 2848 pixels) and exhibit significant variability in terms of focus, illumination, and noise^32^. This instance includes blurred, overexposed, and underexposed samples. To standardize the input size and reduce the computational cost, all images were resized to 224 × 224 pixels before being fed into the CNN models. The labelled dataset was randomly partitioned into training and test subsets in an 80:20 split. The training set contains 2929 images (1485 DR and 1444 No_DR), while the held-out test set includes 733 images (372 DR and 361 No_DR). This breakdown ensures an almost equal distribution of diseased and no-diseased classes in both subsets, enabling fair performance assessment for each category. Figure 3 illustrates the class distribution in the training and testing sets, showing that both partitions preserve the balance between DR and No_DR images overall.

### CLAHE (contrast-limited adaptive histogram equalization)

CLAHE is a local contrast enhancement technique that operates on small, non-overlapping tiles within an image. Within each tile, the histogram is equalized and the contrast is amplified to a limited extent, preventing the over-enhancement of noise and the introduction of unnatural artifacts. Once all the tiles have been processed, bilinear interpolation is applied to merge neighboring regions smoothly and avoid visible grid boundaries^30^.

This behavior makes CLAHE particularly effective for low-contrast images, such as medical images, where subtle local structures must be enhanced without distorting the overall appearance. In practice, CLAHE is primarily controlled by two parameters: the clipLimit, which determines the maximum allowable contrast amplification, and the tileGridSize, which defines the spatial scale of local enhancement. In most implementations (e.g., in common computer vision libraries), CLAHE can be applied to both grayscale and color images. For color images, it is usually applied to the luminance channel only, preserving the original color information while improving local contrast^33^.

The overall CLAHE process can be summarized as follows^30^:

- **Image division**: The input image is divided into small regions (tiles) of a predefined size.
- **Histogram computation**: A histogram is computed for each tile to represent the distribution of grey levels in that region.
- **Histogram clipping**: The histogram of each tile is clipped at a predefined clip limit to restrict peak values and control contrast amplification.
- **Histogram equalization**: The clipped histogram of each tile is equalized to produce locally enhanced pixel values.
- **Image reconstruction**: The equalized pixel values are then used to reconstruct each enhanced tile. All of the enhanced tiles are subsequently merged to create the final, contrast-enhanced image.

**CLAHE algorithm**:


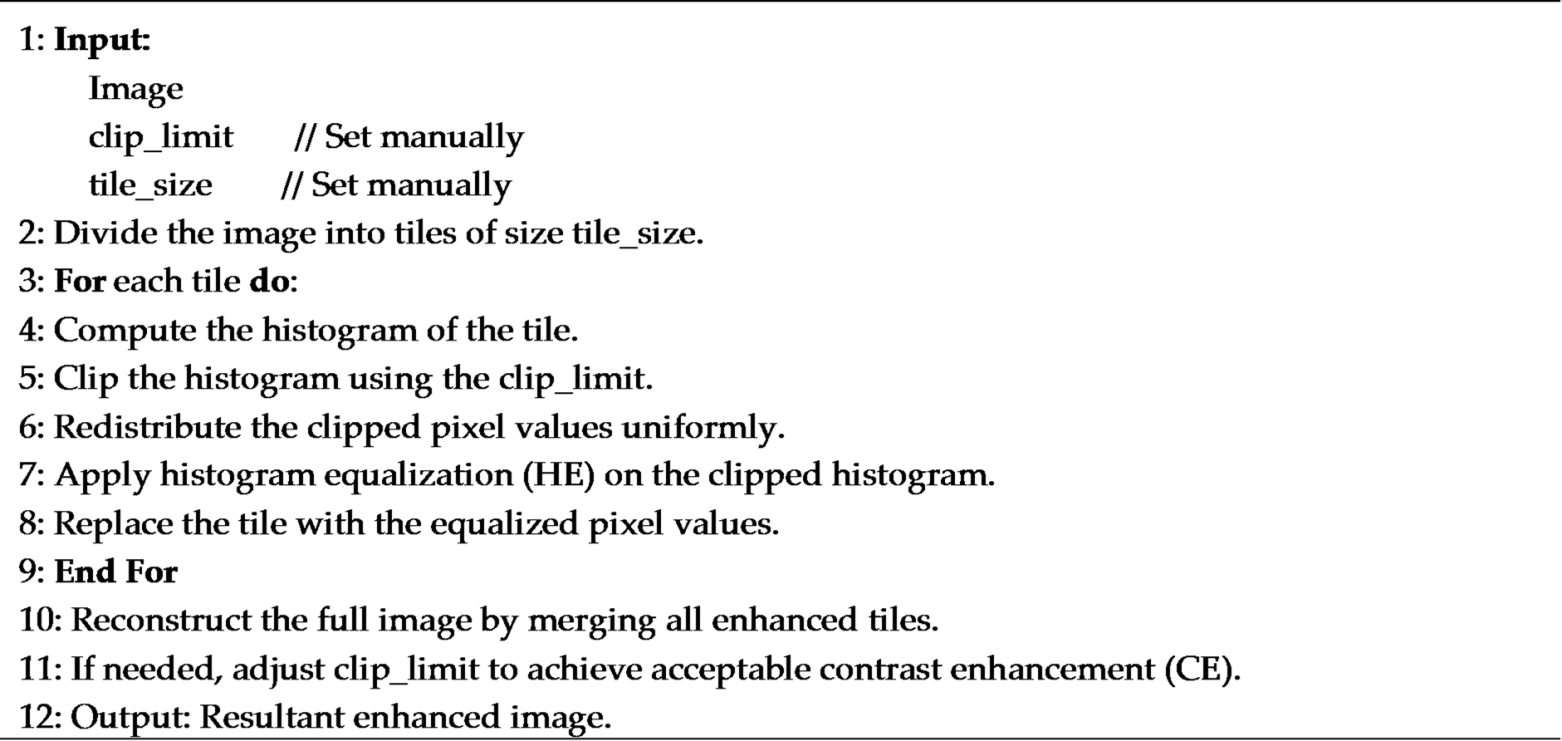


### CNN models

Many researchers, particularly those specializing in medical image processing, use transfer learning (TL) rather than training deep convolutional neural networks (CNNs) from scratch. This is because TL significantly reduces training time and data requirements while improving generalization^34^. In this study, we adopt a multi-stage, deep, ensemble, transfer learning methodology combining feature extraction, pre-trained CNNs, aggregation through ensemble fusion, and a comprehensive performance evaluation. In transfer learning, a model is first trained on a large dataset from a related domain^35^. Then, the model is learned using a smaller, domain-specific dataset. This approach leverages learned low-level and mid-level features from the source domain, thereby avoiding the need for random initialization and substantially reducing the risk of overfitting^36^. A key challenge for CNNs is their reliance on large amounts of annotated training data. The number and depth of model parameters directly influence the minimum dataset size required: networks with more layers require more data to avoid overfitting^37^. In medical imaging, it is often impractical to obtain sufficiently large and diverse annotated datasets due to privacy regulations, the cost of expert annotation, and the rarity of diseases^38^. Transfer learning mitigates these limitations by reusing feature representations learned from large public datasets, such as ImageNet, which makes it particularly valuable for medical applications^39^.

This work selects four state-of-the-art pre-trained CNN architectures: ResNet50, VGG16, VGG19, and DenseNet121. Each model is initialized with ImageNet weights and then retrained on the APTOS 2019 DR dataset for specialization in binary retinal classification.

#### VGG16 and VGG19 models

**Fig. 4:**
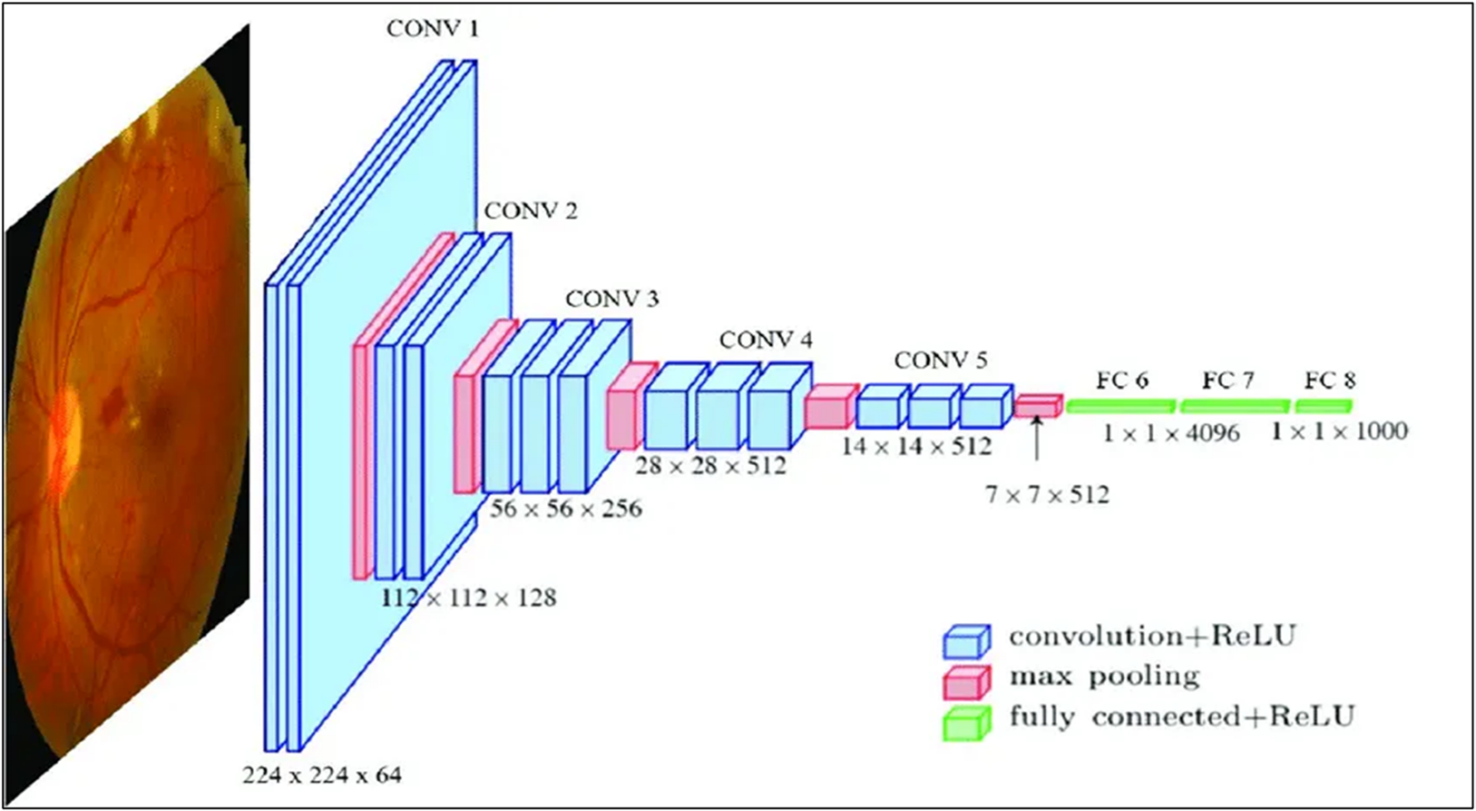
VGGNet architecture for retinal image classification^43^.

In 2014, the Visual Geometry Group (VGG) at the University of Oxford introduced the VGG family of architectures, including variants such as VGG11, VGG13, VGG16, and VGG19 (see Fig. 4)^40^. VGG16 and VGG19 are the most widely adopted versions, particularly in medical imaging applications for the recognition and classification of retinal pathology. VGG16 has 16 learnable convolutional and fully connected layers that are grouped into five convolutional blocks^41^. After that, there are three dense layers. Despite using relatively small 3 × 3 convolutional kernels, VGG16 and VGG19 are computationally intensive and require substantial GPU memory^42^. However, their straightforward architecture and proven efficacy in DR detection make them excellent choices for ensemble learning.

#### DenseNet121 architecture

**Fig. 5:**
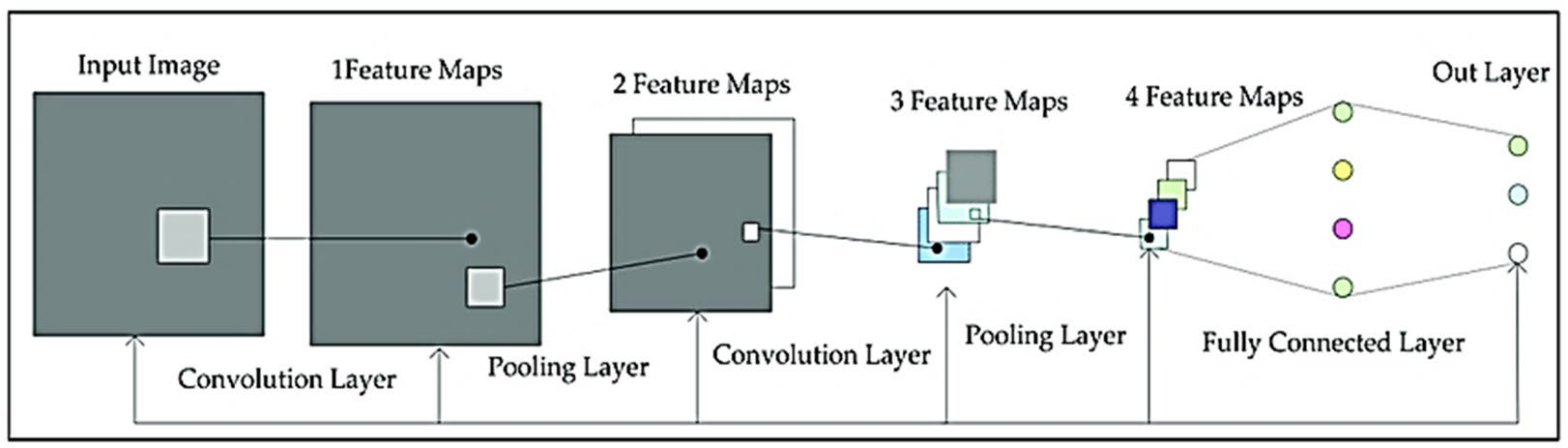
Architecture of the DenseNet121 Model^48^.

DenseNet121 is a densely connected convolutional network that addresses several key challenges in deep learning^44^. It facilitates improved gradient flow through dense skip connections by design, enabling efficient backpropagation during training and reducing the vanishing gradient problem that typically occurs as network depth increases. Its core innovation is that each layer receives inputs from all preceding layers, promoting efficient feature reuse, minimizing feature redundancy, reducing parameter count, and improving computational efficiency^45^. The vanishing gradient problem, whereby error signals decay as they propagate backwards through many layers, is mitigated in DenseNet121 through these dense skip connections, which create direct pathways for gradient flow^46^. Unlike traditional sequential architectures, where information can be lost or diluted as the network deepens, DenseNet121’s dense connectivity pattern ensures that both low- and high-level features are learned and preserved together. This leads to robust representations and improved generalization on medical imaging tasks (Fig. 5)^47^.

#### ResNet50 architecture

**Fig. 6:**
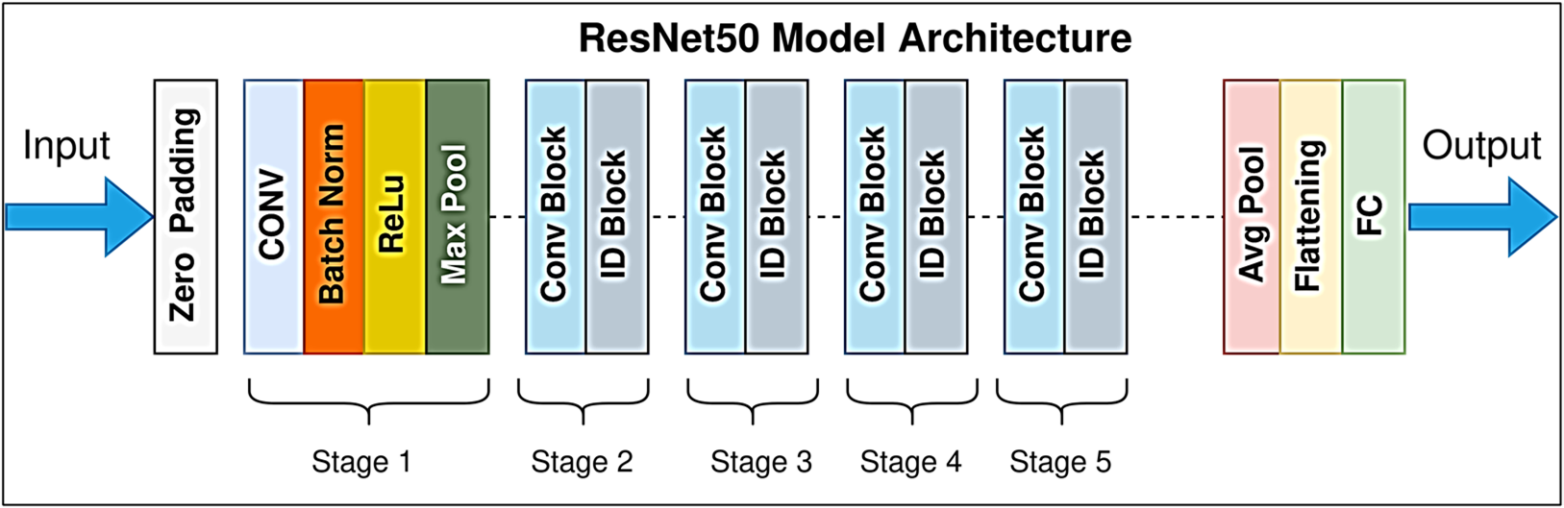
Architecture of the ResNet50 Model^52^.

ResNet (Residual Network) is a revolutionary deep learning architecture that addresses the vanishing gradient problem through skip connections, also known as residual connections or identity mappings^49^. Unlike traditional sequential architectures, where gradients can decay when backpropagating through many layers, ResNet’s skip connections create direct pathways that allow gradients to flow unobstructed through the network. This design enables significantly deeper networks to be trained without performance degradation, making ResNet particularly effective for medical image analysis tasks^50^. ResNet50, a member of the ResNet family, consists of 50 layers organized into five residual blocks (stages), with skip connections spanning multiple layers within and across blocks. Each residual block combines convolutional layers, batch normalization, and ReLU activations, as well as identity shortcuts, which allow the network to learn residual functions rather than the desired mappings directly. This improves convergence speed, reduces overfitting, and enhances feature extraction from retinal fundus images (Fig. 6)^51^.

### Hyperparameter selection and optimization strategy

To ensure fair and stable model comparison, a systematic hyperparameter selection strategy was adopted. Hyperparameters were selected based on empirical evaluations of convergence stability, generalization performance, and computational efficiency. Batch sizes of 64 and 128 were tested to balance gradient stability and memory constraints. Learning rates of 1 × 10^-3^, 1 × 10^-4^, and 1 × 10^-5^ were evaluated using SGD, RMSprop, and Adam optimizers. The Adam optimizer with a learning rate of 1 × 10^-4^ consistently demonstrated faster convergence, reduced testing loss oscillation, and superior testing accuracy across all CNN backbones. Smaller learning rates slowed convergence without measurable accuracy improvement, while larger learning rates caused unstable training behavior.

**Table 2:** Justification of hyperparameter choices used in the experiments.

Parameter	Tested Values	Selected Value	Justification
Batch size	32, 64, 128	64 / 128	Stable gradient updates and efficient training
Learning rate	1e^− 3^, 1e^− 4^, 1e^− 5^	1e^− 4^	Best convergence stability
Optimizer	SGD, RMSprop, Adam	Adam	Faster and smoother convergence
Epochs	30–100	50	Testing performance plateau
Data augmentation	Low / Medium / High	Medium	Reduced overfitting

The number of training epochs was fixed at 50 based on early stopping behavior observed during preliminary experiments, where testing performance saturated beyond this point. Data augmentation intensity and CLAHE parameters were empirically validated to enhance lesion visibility and mitigate overfitting. The selected hyperparameter configuration therefore represents an optimal trade-off between performance stability, generalization capability, and computational efficiency. The evaluated hyperparameter ranges and the selected configuration are summarized in Table 2.

### Ensemble classifier method

Ensemble learning is a method of machine learning that uses the predictions of several separate models to make a better classification decision^53^. Rather than relying on a single model, ensemble methods utilize the complementary strengths and diversity of the base learners to reduce variance, mitigate overfitting, and enhance generalization^54^. In medical imaging tasks such as the detection of diabetic retinopathy, ensemble approaches have consistently outperformed individual models, particularly when the base learners have diverse architectures with different feature extraction capabilities^55^.

**Fig. 7:**
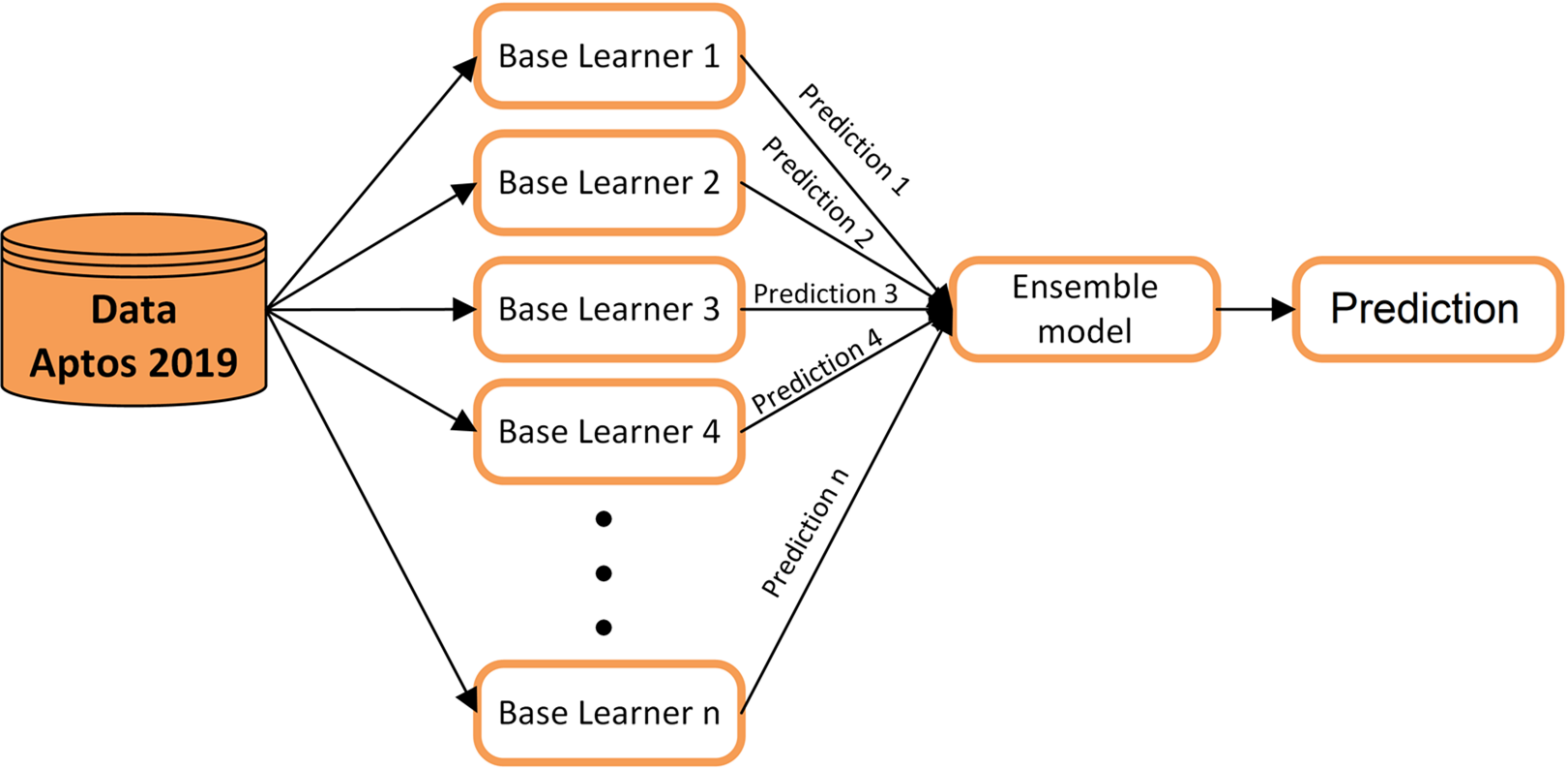
Ensemble learning strategy for combining predictions from multiple models.

In this study, we use four pre-trained CNN architectures (ResNet50, VGG16, VGG19, and DenseNet121) as the base learners. Each model is trained independently on the APTOS 2019 DR dataset, and their individual predictions are then combined using seven complementary ensemble fusion strategies (hard voting, soft voting, weighted soft voting, rank-based fusion, Choquet integral, Sugeno integral, and average logits fusion). These strategies operate at the decision level, aggregating output probabilities or class assignments from all base learners to produce a final ensemble-based DR classification. The ensemble method has a number of benefits: it lowers the chance that any one model’s biases or failure modes will affect the final prediction; it makes the model more robust to changes and noise in retinal photos; and it gives more reliable confidence scores for medical decision support. Figure 7 illustrates the core concept of ensemble learning as applied to our proposed RDE-DR system.

#### Ensemble fusion strategies

The RDE-DR framework employs seven complementary fusion strategies to combine predictions from four base CNN models: ResNet50, VGG16, VGG19, and DenseNet121. Each strategy operates at the decision level, aggregating output probabilities or confidence scores to produce a final binary DR classification.

1. Hard Voting with Threshold Optimization is a majority-rule ensemble method in which each base classifier casts a discrete class vote. The final prediction is assigned to whichever class receives the most votes^56^.

1 $$\:\mathrm{c}\mathrm{l}\mathrm{a}\mathrm{s}\mathrm{s}\left(\mathrm{I}\right)=\mathrm{arg}{max}_{k}\:\sum\:_{j=1}^{m}{\widehat{y}}_{j}=k$$

where $\:1({\stackrel{\prime }{y}}_{j}=k)$ denotes the indicator function, which equals 1 if classifier *j* predicts class *k* and 0 otherwise, *m* is the total number of base classifiers (m = 4 in this study).

With Threshold Optimization:

For binary DR classification, a tunable decision threshold τ ∈ [0,1] is introduced, where τ represents the minimum ensemble probability required to classify an image as DR-positive:

2 $$\:\mathrm{c}\mathrm{l}\mathrm{a}\mathrm{s}\mathrm{s}\left(\mathrm{I}\right)=\left\{\begin{array}{c}1\:\left(DR\right)\:\:\:\:if\:\frac{{\sum\:}_{j=1}^{m}{\widehat{y}}_{i}}{m}\ge\:\tau\:\\\:0\:\left(No\_DR\right)\:\:Otherwise\:\:\:\end{array}\right.$$

Threshold optimization involves adjusting $\:\tau\:$ to maximize a chosen metric (e.g., F1 score or balanced accuracy) on a testing set.

2. Soft Voting with Threshold Optimization.

In soft voting, also known as average probability voting, the predicted probability distributions from all base classifiers are combined by averaging their output probabilities^57^.

3 $$\:{P}_{\mathrm{e}\mathrm{n}\mathrm{s}\mathrm{e}\mathrm{m}\mathrm{b}\mathrm{l}\mathrm{e}}\left(\mathrm{k}\right)=\frac{1}{m}\sum\:_{j=1}^{m}{P}_{j}\left(k\right)$$

where $\:{P}_{j}\left(k\right)$ is the predicted probability of class k from classifier j, and the final class is:

4 $$\:\mathrm{c}\mathrm{l}\mathrm{a}\mathrm{s}\mathrm{s}\left(\mathrm{I}\right)=\mathrm{arg}{max}_{k}{P}_{\mathrm{e}\mathrm{n}\mathrm{s}\mathrm{e}\mathrm{m}\mathrm{b}\mathrm{l}\mathrm{e}}\left(\mathrm{k}\right)$$

With Threshold Optimization:

5 $$\:\mathrm{c}\mathrm{l}\mathrm{a}\mathrm{s}\mathrm{s}\left(\mathrm{I}\right)=\left\{\begin{array}{c}1\:\left(DR\right)\:\:\:\:if\:{P}_{\mathrm{e}\mathrm{n}\mathrm{s}\mathrm{e}\mathrm{m}\mathrm{b}\mathrm{l}\mathrm{e}}\left(1\right)\ge\:\tau\:\\\:0\:\left(No\_DR\right)\:\:\:\:\:\:\:\:\:\:\:Otherwise\:\:\:\end{array}\right.$$

where τ is optimized on testing data to balance sensitivity and specificity.

3. Weighted Soft Voting with Threshold Optimization.

Weighted soft voting builds on the concept of soft voting by assigning a weight $\:{\omega\:}_{j}\in\:\left[\mathrm{0,1}\right]$ to each base classifier j, where $\:{\omega\:}_{j}$ reflects the classifier’s relative reliability or testing performance. The weights satisfy the normalization constraint $\:\sum\:_{j=1}^{m}\:{\omega\:}_{j}=1$^58^.

6 $$\:{P}_{\mathrm{w}\mathrm{e}\mathrm{i}\mathrm{g}\mathrm{h}\mathrm{t}\mathrm{e}\mathrm{d}}\left(\mathrm{k}\right)=\frac{{\sum\:}_{j=1}^{m}{\omega\:}_{j\:}{\cdot \:\:P}_{j}\left(k\right)}{{\sum\:}_{j=1}^{m}{\omega\:}_{j\:}}$$

Weights can be assigned based on the accuracy, area under the curve (AUC), or F1-score of individual classifiers on testing data:

7 $$\:{\omega\:}_{j\:}=\frac{{Score}_{j}}{{\sum\:}_{j=1}^{m}{Score}_{j}}$$

Final Classification with Threshold:

8 $$\:\mathrm{c}\mathrm{l}\mathrm{a}\mathrm{s}\mathrm{s}\left(\mathrm{I}\right)=\left\{\begin{array}{c}1\:\left(DR\right)\:\:\:\:if\:{P}_{\mathrm{w}\mathrm{e}\mathrm{i}\mathrm{g}\mathrm{h}\mathrm{t}\mathrm{e}\mathrm{d}}\left(1\right)\ge\:\tau\:\\\:0\:\left(No\_DR\right)\:\:\:\:\:\:\:\:\:\:\:Otherwise\:\:\:\end{array}\right.$$

4. Rank-Based Fusion with Threshold Optimization.

A rank is assigned to each classifier’s output score^59^. These ranks are then aggregated to produce the final decision.

**Figure:**
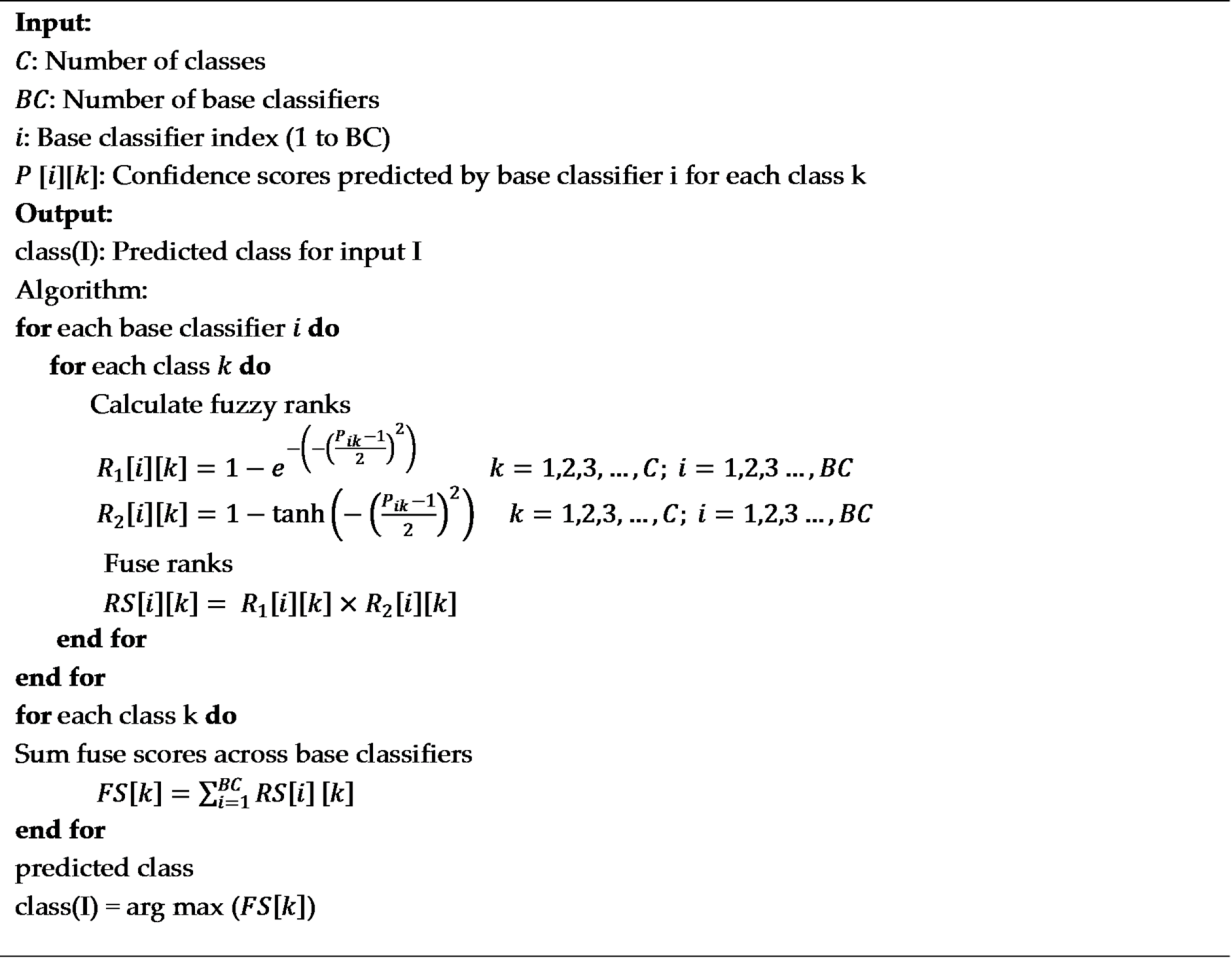
**Algorithm 1** Fuzzy Rank-Based Fusion Algorithm.

5. Choquet-like Integral with Threshold Optimization.

The Choquet integral is a fuzzy aggregation operator that accounts for interactions among classifiers through a fuzzy measure µ. where µ(S) ∈ [0,1] quantifies the importance of any subset S of classifiers. The measure satisfies the boundary conditions µ(∅) = 0 and µ(N) = 1, where N denotes the full set of classifiers^60^.

Let $\:{a}_{1},\:{a}_{2},\dots\:,{a}_{n}\:$denote the classifier confidence scores sorted in non-decreasing order. The Choquet integral with respect to the fuzzy measure µ is defined as:

9 $$\:{C}_{\mu\:}({a}_{1},\:{a}_{2},\dots\:,{a}_{n})=\sum\:_{i=1}^{n}\left({a}_{\mathrm{i}}-{a}_{\mathrm{i}-1}\right)\mu\:\left({A}_{i}\right)$$

where $\:{a}_{0}=0\:$and $\:{A}_{i}$= {$\:i,\:i+1,\dots\:,n\}$ represents the set of classifiers with greater than or equal to scores $\:{a}_{\mathrm{i}}$.

For binary diabetic retinopathy (DR) classification with four classifiers, the predicted confidence scores are first sorted: $\:{P}_{\left(1\right)}\le\:{P}_{\left(2\right)}\le\:{P}_{\left(3\right)}\le\:{P}_{\left(4\right)}$

A fuzzy measure µ is then defined over all subsets of classifiers (typically learned from testing data or manually assigned). The Choquet integral is computed as:

10 $$\:{C}_{\mu\:}=\left({P}_{\left(1\right)}-0\right)\:\mu\:\left(\left\{\mathrm{1,2},\mathrm{3,4}\right\}\right)+\left({P}_{\left(2\right)}-{P}_{\left(1\right)}\right)\:\mu\:\left(\left\{\mathrm{2,3},4\right\}\right)+\dots\:$$

The final decision is obtained using a threshold τ:

11 $$\:\mathrm{c}\mathrm{l}\mathrm{a}\mathrm{s}\mathrm{s}\left(\mathrm{I}\right)=\left\{\begin{array}{c}1\:\:\left(DR\right)\:\:\:\:if\:{C}_{\mu\:}\ge\:\tau\:\:\:\:\:\:\:\:\:\:\:\:\:\:\:\:\:\\\:0\:\:\:\:\:\:\:\:\:\:\left(No\_DR\right)\:\:\:Otherwise\end{array}\right.$$

6. Sugeno Integral with Threshold Optimization.

Another fuzzy aggregation operator that combines classifier outputs is the Sugeno integral, which uses a Sugeno $\:{\uplambda\:}-\mathrm{f}\mathrm{u}\mathrm{z}\mathrm{z}\mathrm{y}\:$measure^61^.

Step 1: Initialize Sugeno $\:{\uplambda\:}-\mathrm{f}\mathrm{u}\mathrm{z}\mathrm{z}\mathrm{y}\:$measure.

The Sugeno λ-fuzzy measure $\:{\mu\:}_{\lambda\:}$ is defined recursively, where λ∈ (− 1, ∞), λ ≠ 0, is an interaction parameter that controls the degree of complementarity (λ < 0) or redundancy (λ > 0) among classifiers. For a single classifier$\:\:i$, the fuzzy measure is defined as:

12 $$\:{\mu\:}_{\lambda\:}\left(\right\{i\left\}\right)={g}_{i}$$

where $\:{g}_{i}$ ∈ [0,1] denotes the fuzzy density (importance weight) of classifier $\:i$. The values $\:{g}_{i}$​ are normalized and typically estimated from testing performance metrics such as classification accuracy or AUC.

For multiple classifiers, the Sugeno $\:{\uplambda\:}-\mathrm{m}\mathrm{e}\mathrm{a}\mathrm{s}\mathrm{u}\mathrm{r}\mathrm{e}\:$satisfies:

13 $$\:{\mu\:}_{\lambda\:}\left(\mathrm{A}\cup\:\mathrm{B}\right)={\mu\:}_{\lambda\:}\left(\mathrm{A}\right)+{\mu\:}_{\lambda\:}\left(\mathrm{B}\right)+{\uplambda\:}\:\cdot \:\:{\mu\:}_{\lambda\:}\left(\mathrm{A}\right)\:\cdot \:\:{\mu\:}_{\lambda\:}\left(\mathrm{B}\right)$$

Step 2: Solve for $\:\lambda\:$

14 $$\:1+\lambda\:=\prod\:_{i=1}^{n}{(1+g}_{i})$$

Solve this equation numerically to obtain $\:\lambda\:.$.

Step 3: Compute $\:\mu\:$ for All Subsets.

Using the recursive formula and the computed $\:\lambda\:$, calculate $\:{\mu\:}_{\lambda\:}\left(S\right)$ for all relevant subsets $\:S$.

Step 4: Compute Sugeno Integral.

Sort the classifier outputs in non-decreasing order: $\:{a}_{1}\le\:{a}_{2}\le\:\dots\:\le\:{a}_{n}.$

15 $$\:{S}_{\mu\:}({a}_{1},\dots\:,{a}_{n})={max}_{i=1}^{n}[\mathrm{m}\mathrm{i}\mathrm{n}({a}_{i},\:{\mu\:}_{\lambda\:}\left({A}_{i}\right)\left)\right]$$

where $\:{A}_{i}$= {$\:i,\:i+1,\dots\:,n\}$ is the set of classifiers with scores $\:{\ge\:a}_{i}.$.

**Figure:**
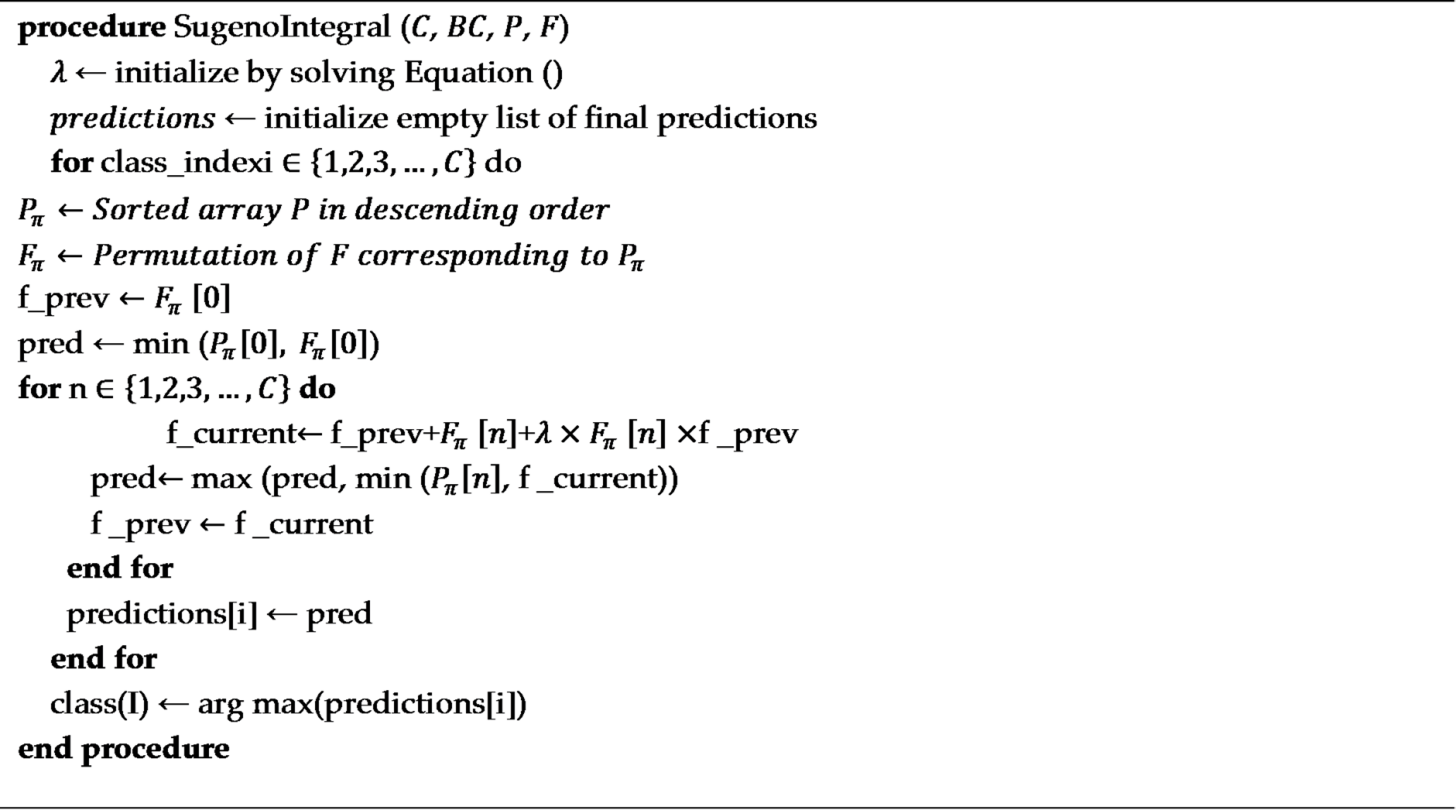
**Algorithm 2** Sugeno integral.

With Threshold Optimization:

16 $$\:\mathrm{c}\mathrm{l}\mathrm{a}\mathrm{s}\mathrm{s}\left(\mathrm{I}\right)=\left\{\begin{array}{c}1\:\:\left(DR\right)\:\:\:\:if\:{S}_{\mu\:}\ge\:\tau\:\:\:\:\:\:\:\:\:\:\:\:\:\:\:\:\:\\\:0\:\:\:\:\:\:\:\:\:\:\left(No\_DR\right)\:\:\:Otherwise\end{array}\right.$$

7. Average Logits Fusion.

This algorithm operates on the raw logits (i.e., unnormalized scores) from each classifier before the SoftMax function is applied, producing the final probability distribution^62^.

17 $$\:{L}_{avg}=\frac{1}{m}\sum\:_{j=1}^{m}{L}_{j}$$

where $\:{L}_{j}$ ∈ ℝ^2^ denotes the logits vector, defined as the raw, unnormalized output scores produced by classifier $\:j$ prior to the softmax activation. The components of $\:{L}_{j}=[{l}_{j,0}\text{},{l}_{j,1}\text{}\text{}]$ correspond to the No_DR and DR classes, respectively.

Then apply SoftMax:

18 $$\:{P}_{\mathrm{e}\mathrm{n}\mathrm{s}\mathrm{e}\mathrm{m}\mathrm{b}\mathrm{l}\mathrm{e}}\left(\mathrm{k}\right)=\frac{\mathrm{e}\mathrm{x}\mathrm{p}\left({L}_{avg,k}\right)}{\sum\:_{k}\mathrm{e}\mathrm{x}\mathrm{p}\left({L}_{avg,k}\right)}$$

**Final Classification**:

19 $$\:\mathrm{c}\mathrm{l}\mathrm{a}\mathrm{s}\mathrm{s}\left(\mathrm{I}\right)=\left\{\begin{array}{c}1\:\:\left(DR\right)\:\:\:\:if\:{P}_{\mathrm{e}\mathrm{n}\mathrm{s}\mathrm{e}\mathrm{m}\mathrm{b}\mathrm{l}\mathrm{e}}\left(1\right)\ge\:\tau\:\:\:\:\:\:\:\:\:\:\:\:\:\:\:\:\:\\\:0\:\:\:\:\:\:\:\:\:\:\left(No\_DR\right)\:\:\:Otherwise\end{array}\right.$$

#### Threshold optimization

Threshold optimization is a post-hoc calibration step that is applied after ensemble predictions have been computed^63^. Rather than using the default decision boundary of 0.5, a custom threshold (τ) is optimized on the testing set to maximize a specific performance metric.

Threshold Optimization Procedure^64^:

1. Generate predictions on testing data using each fusion strategy (output: probability scores for class 1, i.e., DR).
2. Sweep threshold values $\:\tau\:\in\:[0,\:1]$ in small increments (e.g., 0.01).
3. For each threshold, compute performance metrics:

$$\:\mathrm{A}\mathrm{c}\mathrm{c}\mathrm{u}\mathrm{r}\mathrm{a}\mathrm{c}\mathrm{y},\:\mathrm{S}\mathrm{e}\mathrm{n}\mathrm{s}\mathrm{i}\mathrm{t}\mathrm{i}\mathrm{v}\mathrm{i}\mathrm{t}\mathrm{y},\:\mathrm{S}\mathrm{p}\mathrm{e}\mathrm{c}\mathrm{i}\mathrm{f}\mathrm{i}\mathrm{c}\mathrm{i}\mathrm{t}\mathrm{y},\:\mathrm{F}1-\mathrm{S}\mathrm{c}\mathrm{o}\mathrm{r}\mathrm{e},\:\mathrm{B}\mathrm{a}\mathrm{l}\mathrm{a}\mathrm{n}\mathrm{c}\mathrm{e}\mathrm{d}\:\mathrm{A}\mathrm{c}\mathrm{c}\mathrm{u}\mathrm{r}\mathrm{a}\mathrm{c}\mathrm{y}=\:\frac{\mathrm{S}\mathrm{e}\mathrm{n}\mathrm{s}\mathrm{i}\mathrm{t}\mathrm{i}\mathrm{v}\mathrm{i}\mathrm{t}\mathrm{y}+\mathrm{S}\mathrm{p}\mathrm{e}\mathrm{c}\mathrm{i}\mathrm{f}\mathrm{i}\mathrm{c}\mathrm{i}\mathrm{t}\mathrm{y}\:}{2}$$

20 $$\:{\tau\:}^{\mathrm{*}}={arg\:}\underset{\tau\:\in\:[0,\:1]}{\mathrm{max}}\mathrm{F}1\left({\uptau\:}\right)\:$$

where $\:{\tau\:}^{\mathrm{*}}$ denotes the threshold that maximizes the F1-score on the testing set.

Apply to test data using the optimal threshold:

21 $$\:\mathrm{c}\mathrm{l}\mathrm{a}\mathrm{s}\mathrm{s}\left(\mathrm{I}\right)=\left\{\begin{array}{c}1\:\:\left(DR\right)\:\:\:\:if\:{P}_{\mathrm{e}\mathrm{n}\mathrm{s}\mathrm{e}\mathrm{m}\mathrm{b}\mathrm{l}\mathrm{e}}\left(I\right)\ge\:{\tau\:}^{\mathrm{*}}\:\:\:\:\:\:\:\:\:\:\:\:\:\:\:\\\:0\:\:\:\:\:\:\:\:\:\:\left(No\_DR\right)\:\:\:Otherwise\end{array}\right.$$

### Overfitting prevention

Several strategies were employed to mitigate overfitting and improve generalization. All CNN models were initialized using ImageNet pre-trained weights and retrained on the target dataset, reducing reliance on limited training samples. Data augmentation was applied online during training, including random rotations, flips, scaling, and brightness variations, to increase data diversity. Dropout layers and weight decay regularization were used to prevent feature co-adaptation. Batch normalization stabilized gradient propagation and improved convergence. Early stopping based on testing loss was applied to avoid over-training, and the best-performing model weights were retained using checkpointing. Finally, ensemble fusion aggregated predictions from multiple independently trained models, reducing variance and improving robustness.

## Results

This section provides a thorough evaluation of the RDE-DR framework for binary diabetic retinopathy classification. The performance of individual base learners (ResNet50, VGG16, VGG19, and DenseNet121) is evaluated, as is that of all seven ensemble fusion strategies (hard voting, soft voting, weighted soft voting, rank-based fusion, Choquet integral, Sugeno integral, and average logits fusion), each with threshold optimization. The results, which are reported on the held-out test set, use relevant metrics: accuracy, precision, recall (sensitivity), specificity, F1-score, and area under the receiver operating characteristic curve (ROC-AUC). Additionally, we provide confusion matrices and comparative analyses with state-of-the-art methods from the literature to contextualize the performance of RDE-DR.

The APTOS 2019 dataset was divided into training (2,929 images) and test (733 images) sets using an 80:20 split, with balanced class distributions in both subsets (see Fig. 3). All results were computed on the test set, ensuring no information leaked from the training data.

### Experimental protocol

The experimental analysis of diabetic retinopathy classification was conducted on a local workstation dedicated to high-performance deep learning tasks. This setup was optimized to efficiently handle extensive image processing and training workloads. Table 3 reports the hardware configuration of our local workstation used.

The experiments were implemented using Python 3.7 with the integrated Jupyter Notebook interface on the local machine. This facilitated consistent code development, model training and visualization in a single environment. This environment supports an efficient, reproducible deep learning workflow, facilitating the development of models, the tuning of hyperparameters, and the evaluation of models for the classification of diabetic retinopathy.

### Evaluation metrics

Selecting suitable evaluation metrics is essential for reliably assessing model performance in the classification of diabetic retinopathy. Metrics such as accuracy, recall, precision, F1_score, and specificity are frequently employed, as each provides a distinct viewpoint regarding various aspects of predictive capability. The mathematical definitions of these metrics are provided in Eqs. (22–26), which serve as the foundation for the performance analysis conducted in this study.

**Table 3:** Hardware configuration of the local workstation used for diabetic retinopathy classification experiments.

Component	Specification
**GPU**	**NVIDIA GeForce RTX 3090**
GPU Driver	Version: 32.0.15.6094
DirectX	Version 12 (feature level 12.1)
GPU Location	PCI bus 1, device 0, function 0
Total GPU Memory	55.9 GB
**CPU**	**13th Gen Intel® Core™ i7-13700 K**
CPU Base Clock	3.40 GHz
CPU Cores	16 (24 logical processors)
CPU Caches	L1: 1.4 MB, L2: 24 MB, L3: 30 MB
CPU Operating Speed	~ 3.81 GHz during experiments
Virtualization	Enabled
**Memory**	**64 GB DDR4 RAM at 3200 MT/s**
RAM Slots	Fully populated (4 of 4 DIMM)

- Accuracy: is defined as the ratio of correctly predicted instances to the overall number of cases^65^.

22 $$\:\mathrm{A}\mathrm{c}\mathrm{c}\mathrm{u}\mathrm{r}\mathrm{a}\mathrm{c}\mathrm{y}\:\left(\mathrm{A}\mathrm{c}\mathrm{c}\right)=\frac{\left(\mathrm{T}\:\mathrm{P}\:+\:\mathrm{T}\:\mathrm{N}\right)}{\left(\mathrm{T}\:\mathrm{P}\:+\:\mathrm{T}\:\mathrm{N}\:+\:\mathrm{F}\mathrm{P}\:+\:\mathrm{F}\:\mathrm{N}\right)}\mathrm{*}\:100\%$$

- Recall: also referred to as sensitivity, quantifies the classifier’s ability to correctly identify all actual positive cases within the dataset. It represents the proportion of true positive instances that are accurately detected by the model^50^.

23 $$\:\mathrm{R}\mathrm{e}\mathrm{c}\mathrm{a}\mathrm{l}\mathrm{l}\:\left(\mathrm{S}\mathrm{e}\mathrm{n}\mathrm{s}\mathrm{i}\mathrm{t}\mathrm{i}\mathrm{v}\mathrm{i}\mathrm{t}\mathrm{y}\right)=\frac{\mathrm{T}\:\mathrm{P}}{\left(\mathrm{T}\:\mathrm{P}\:+\:\mathrm{F}\:\mathrm{N}\right)}\:\mathrm{*}100\%\:$$

- True Negative Rate (TNR): is a metric that quantifies the precision of a system’s negative identification, calculated as the ratio of true negative instances that are correctly identified^66^.

24 $$\:\mathrm{T}\mathrm{N}\mathrm{R}\:\left(\mathrm{S}\mathrm{p}\mathrm{e}\mathrm{c}\mathrm{i}\mathrm{f}\mathrm{i}\mathrm{c}\mathrm{i}\mathrm{t}\mathrm{y}\right)=\frac{\mathrm{T}\:\mathrm{N}\:}{\left(\mathrm{T}\:\mathrm{N}\:+\:\mathrm{F}\mathrm{P}\:\right)}\mathrm{*}\:100\%$$

- Precision: Precision quantifies the classifier’s ability to correctly identify only the relevant positive instances. It represents the proportion of predicted positive cases that are actually true positives^67^.

25 $$\:\mathrm{P}\mathrm{r}\mathrm{e}\mathrm{c}\mathrm{i}\mathrm{s}\mathrm{i}\mathrm{o}\mathrm{n}\:\left(\mathrm{P}\mathrm{r}\mathrm{e}\right)=\frac{\mathrm{T}\:\mathrm{P}}{\left(\mathrm{T}\:\mathrm{P}\:+\:\mathrm{F}\mathrm{P}\:\right)}\mathrm{*}\:100\%$$

F1_score: is a metric that quantifies the balance between precision and recall by calculating their harmonic mean. This score considers both false positives and false negatives, providing a single value that reflects the trade-off between correctly identified positive cases and errors made by the classifier^68^.

26 $$\:\mathrm{F}{1}_{\mathrm{s}\mathrm{c}\mathrm{o}\mathrm{r}\mathrm{e}}=\frac{2\:\mathrm{*}\:\mathrm{P}\mathrm{r}\mathrm{e}\mathrm{c}\mathrm{i}\mathrm{s}\mathrm{i}\mathrm{o}\mathrm{n}\:\mathrm{*}\:\mathrm{R}\mathrm{e}\mathrm{c}\mathrm{a}\mathrm{l}\mathrm{l}}{\left(\mathrm{P}\mathrm{r}\mathrm{e}\mathrm{c}\mathrm{i}\mathrm{s}\mathrm{i}\mathrm{o}\mathrm{n}\:+\:\mathrm{R}\mathrm{e}\mathrm{c}\mathrm{a}\mathrm{l}\mathrm{l}\right)}\mathrm{*}\:100\%$$

All training, optimization, and data augmentation hyperparameters used in this study are summarized in Table 4.

**Table 4:** Summary of hyperparameters used in experiments.

Category	Hyperparameter	Value
Training	Batch size	8, 16, 32, 64, 128
	Number of epochs	50
	Early stopping patience	10
	Loss function	Binary cross-entropy
Optimization	Optimizer	Adam
	Learning rate	10^− 3^, 10^− 4^, 10^− 5^
	Weight decay	10^− 5^
Augmentation and preprocessing	Input image size	224 × 224 pixels
	Rotation	± 15°
	Horizontal flip	Yes
	CLAHE clip limit	2.0
	CLAHE tile grid size	8 × 8
Transfer learning	Pre-trained weights	ImageNet

### Results of individual classification models

Table 5 presents the performance of the four retrained CNN architectures (VGG16, VGG19, DenseNet121, and ResNet50) evaluated on the held-out APTOS 2019 test set (733 images). using the training configuration detailed in Table 4. All models achieved high and tightly clustered performance, with accuracies ranging from 97.95% to 98.64% and AUC values above 99.48%. This narrow variation suggests that performance gains are primarily driven by transfer learning, CLAHE preprocessing, and systematic hyperparameter optimization rather than architectural differences.

VGG16 achieved the highest accuracy (98.64%) with a recall of 99.19% and only three false negatives, indicating strong sensitivity for DR screening. VGG19 produced comparable accuracy (98.36%) and one of the highest AUC values (99.83%), reflecting stable discrimination across thresholds. DenseNet121 and ResNet50 demonstrated similarly balanced behavior, with accuracies of 98.36% and 98.23% and AUC values of 99.59% and 99.63%, respectively, supported by low misclassification rates in their confusion matrices. Overall, the four architectures exhibit consistent and robust performance, establishing a stable baseline for subsequent ensemble fusion analysis.

**Table 5:** Performance metrics of classification models on the APTOS 2019 dataset including hyper-parameter settings and confusion matrices.

Models	Hyper-parameter				Evaluation Metrics					
	**Batch size**	**Epochs**	**Optimizers**	**learning rate**	**Accuracy (%)**	**Precision (%)**	**Recall (%)**	**F1-Score (%)**	**AUC (%)**	**Matrix of confusion**
VGG16	**64**	**50**	**Adam**	**10** ^**− 4**^	**98.64**	**98.14**	**99.19**	**98.66**	**99.70**	**[[354**,** 7]**,** [3**,** 369]]**
	128	50	Adam	10^− 4^	98.50	97.88	99.19	98.53	99.85	[[353, 8], [3, 369]]
	16	50	Adam	10^− 5^	98.23	98.12	98.39	98.26	99.60	[[352, 7], [6, 369]]
VGG19	**128**	**50**	**Adam**	**10** ^**− 4**^	**98.36**	**97.62**	**99.19**	**98.40**	**99.83**	**[[352**,** 9]**,** [3**,** 369]]**
	64	50	Adam	10^− 4^	98.09	97.11	99.19	98.14	99.83	[[350, 11], [3, 369]]
	8	50	Adam	10^− 5^	97.95	97.10	98.92	98.00	99.48	[[350, 11], [4, 368]]
DenseNet-121	**128**	**50**	**Adam**	**10** ^**− 4**^	**98.36**	**97.87**	**98.92**	**98.40**	**99.59**	**[[353**,** 8]**,** [4**,** 368]]**
	64	50	Adam	10^− 4^	98.09	97.61	98.66	98.13	99.56	[[352, 9], [5, 367]]
	32	50	Adam	10^− 4^	98.09	97.35	98.92	98.13	99.74	[[351, 10], [4, 368]]
Res Net-50	**128**	**50**	**Adam**	**10** ^**− 4**^	**98.23**	**97.61**	**98.92**	**98.26**	**99.63**	**[[352**,** 9]**,** [4**,** 368]]**
	64	50	Adam	10^− 4^	98.09	97.61	98.66	98.13	99.51	[[352, 9], [5, 367]]
	32	50	Adam	10^− 4^	97.95	97.60	98.39	97.99	99.59	[[352, 9], [6, 366]]

All four models exhibit consistently strong performance, with VGG16 and VGG19 achieving the highest recall—an essential criterion in large-scale screening applications to minimize missed DR cases. The uniformly low false negative rates (3–4 misclassified DR cases out of 372) highlight the reliability of the retrained models for automated detection tasks. The close agreement across architectures indicates that transfer learning, CLAHE preprocessing, and optimized hyperparameters contribute more substantially to performance than architectural differences alone.

**Fig. 8:**
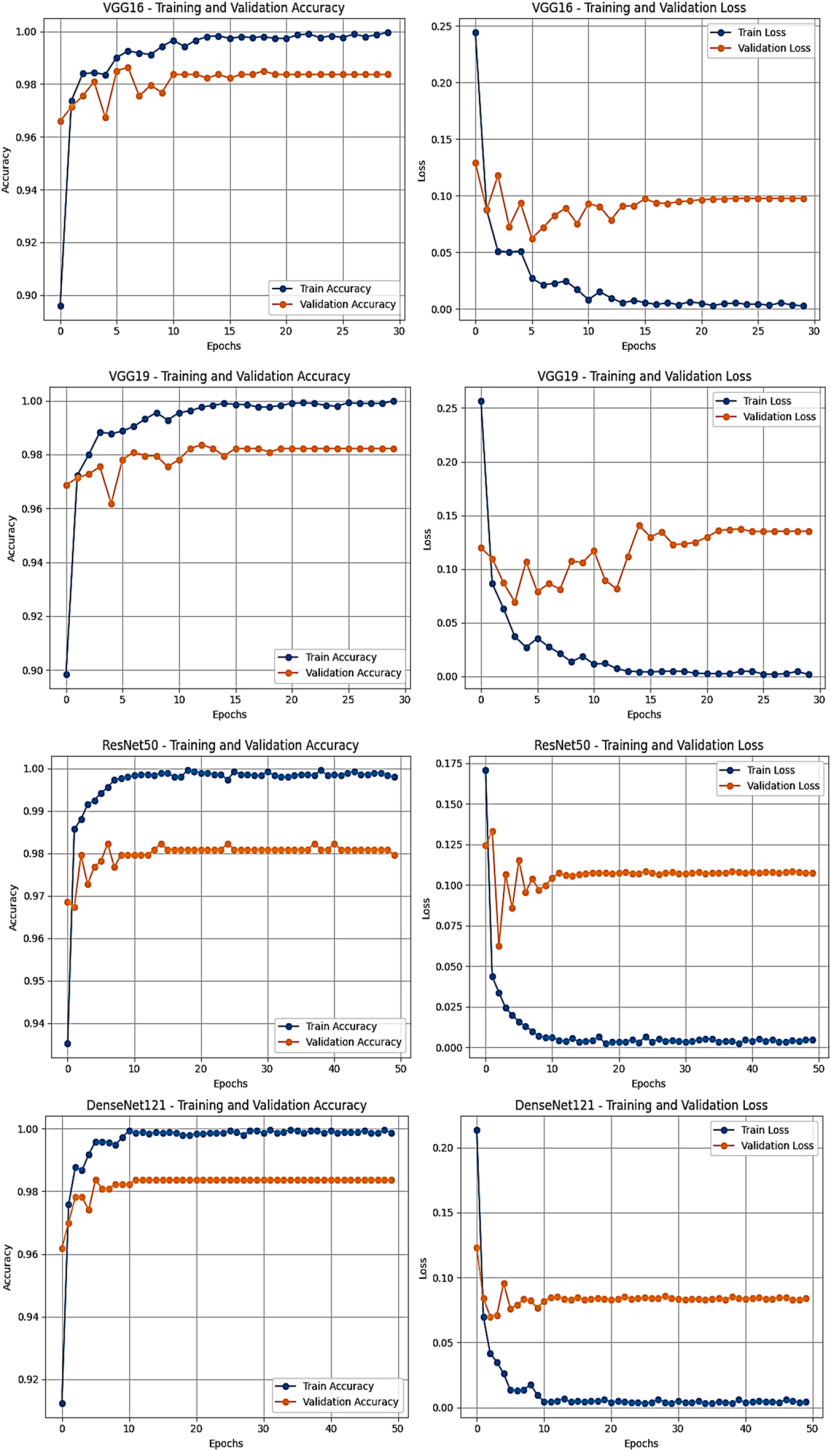
Training and testing accuracy and loss curves for all base CNN models (ResNet50, VGG16, VGG19, DenseNet121) trained on the CLAHE-enhanced APTOS 2019 dataset.

**Fig. 9:**
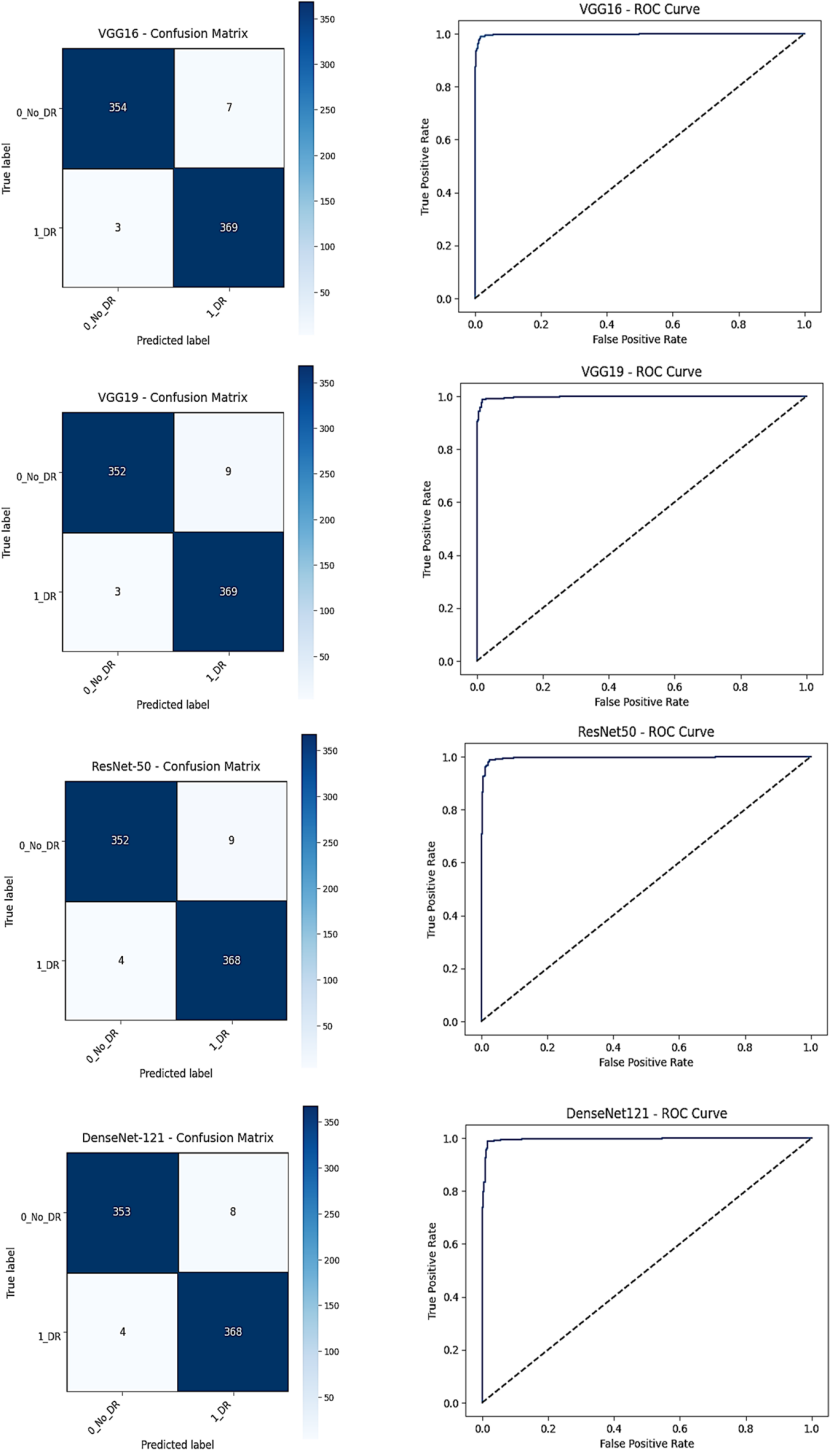
Confusion matrices and ROC curves for all base CNN models (ResNet50, VGG16, VGG19, DenseNet121) on the APTOS 2019 test set.

Figures 8 and 9 further confirm these findings. The training and validation curves demonstrate stable convergence with minimal divergence, suggesting effective overfitting control. The confusion matrices and ROC curves corroborate the high discriminative capacity of all models, with near-perfect class separation on the test set.

### Ensemble fusion results and comprehensive analysis

Table 6 summarizes the performance of the seven ensemble fusion strategies evaluated on the APTOS 2019 test set (733 images). Overall, the ensembles demonstrate consistently high and stable performance across all evaluation metrics. The nearly identical results obtained with hard, soft, and weighted soft voting indicate strong methodological robustness. This convergence across different aggregation mechanisms suggests that performance improvements arise primarily from complementary feature representations learned by the base models rather than from fusion-specific optimization effects.

**Table 6:** Performance metrics of ensemble fusion methods on APTOS 2019 test set.

Fusion method	Accuracy (%)	Precision (%)	Recall (%)	F1-Score (%)	AUC (%)
Hard voting	98.64	98.40	98.92	98.66	99.78
Soft voting	98.64	98.40	98.92	98.66	99.78
Weighted soft voting	98.64	98.40	98.92	98.66	99.79
Rank-based fusion	98.50	98.13	98.92	98.53	99.80
Sugeno integral	97.41	95.61	99.46	97.50	98.13
Choquet-like integral	98.23	97.87	98.66	98.26	99.80
Average logits fusion	98.50	98.13	98.92	98.53	99.79

ROC–AUC analysis further confirms the discriminative strength of the proposed approach. While individual CNNs already achieved high AUC values (99.59%–99.83%), ensemble strategies maintained or slightly enhanced this performance. The highest AUC (99.80%) was obtained with rank-based and Choquet-like fusion methods, indicating excellent threshold-independent separability. In contrast, the Sugeno integral method yielded comparatively lower performance (AUC = 98.13%), suggesting reduced aggregation effectiveness under this formulation.

Generally, ensemble fusion preserves the strong baseline discrimination of individual models while providing marginal but consistent stability improvements, supporting its suitability for robust automated DR detection.

**Fig. 10:**
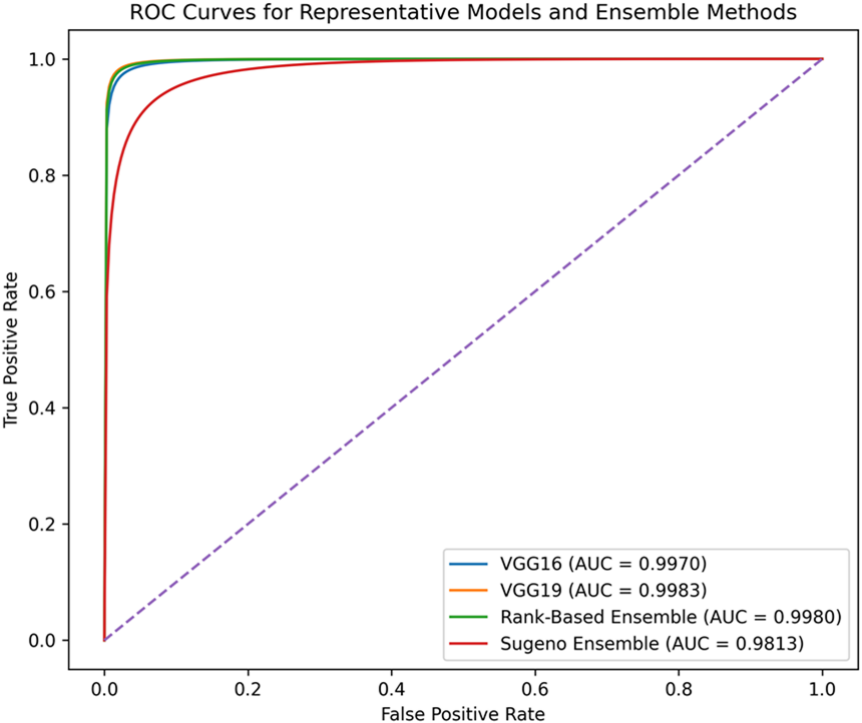
ROC curves for representative individual CNN models and the best-performing ensemble configuration on the APTOS 2019 dataset.

Figure 10 presents the ROC curves of representative individual CNN models and the best-performing ensemble configuration. The ensemble curve closely overlaps with—and slightly dominates—the strongest individual models across most operating regions, confirming stable threshold-independent discrimination. The best ensemble achieves a ROC–AUC of 99.80%, indicating excellent class separability and consistent calibration behavior. In contrast, the Sugeno integral method shows visible degradation, aligning with its lower AUC value reported in Table 6.

Altogether, the ROC analysis corroborates that ensemble fusion preserves the high discriminative capacity of the base models while providing improved stability across decision thresholds.

**Fig. 11:**
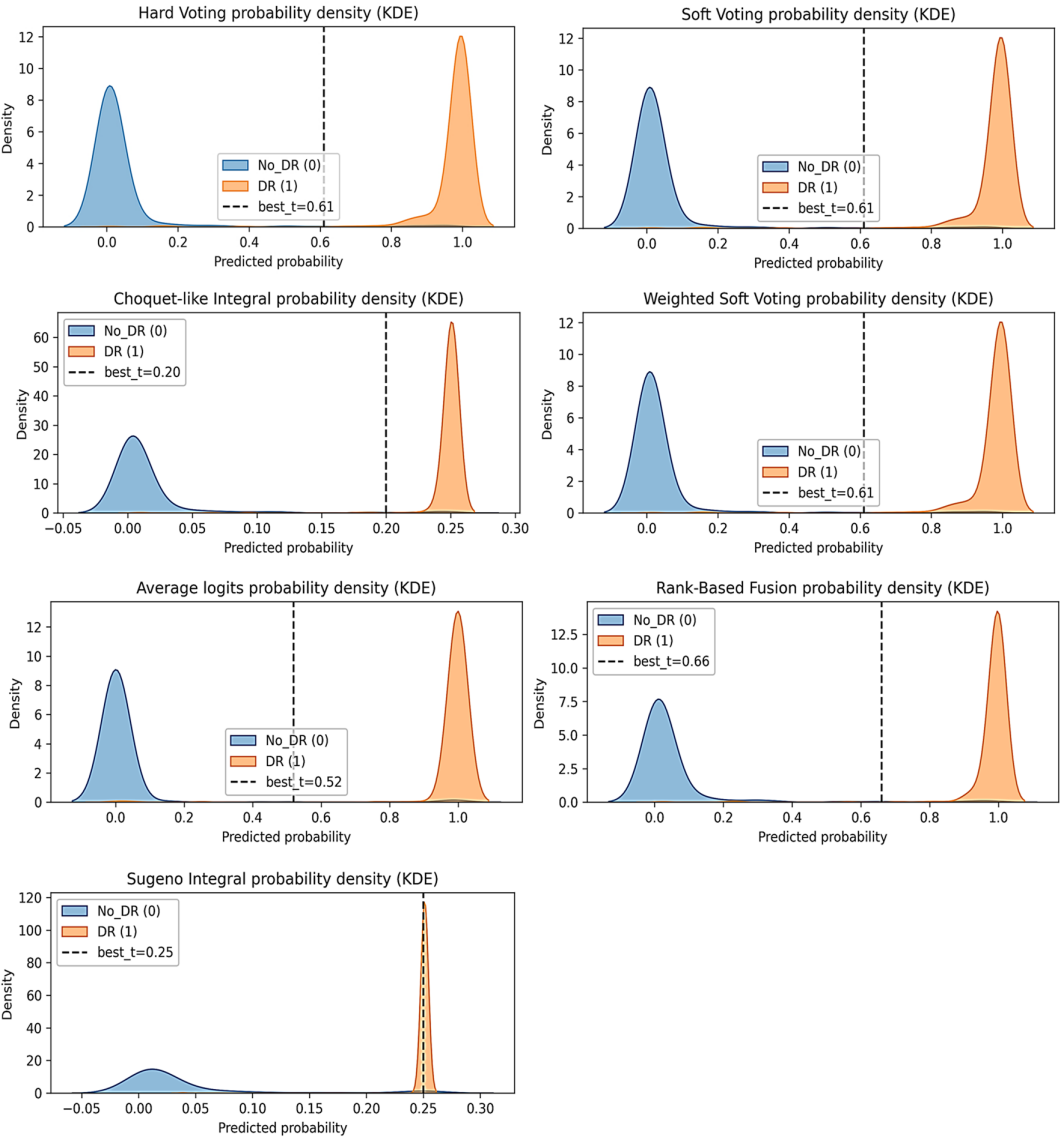
Ensemble fusion method probability density distributions and optimized decision thresholds (KDE plots for all seven methods).

Figure 11 shows kernel density estimation (KDE) plots that show the predicted probability distributions for each ensemble fusion method applied to the test set. Each subplot shows the No_DR class in blue on the left and the DR class in orange on the right, with the optimal decision threshold in black dashed lines, determined through testing set optimization. Hard voting, soft voting, and weighted soft voting demonstrate exceptional class separation with thresholds at 0.61; rank-based fusion employs a threshold of 0.66; and average logits fusion uses 0.52. The fuzzy integral methods (Choquet-like: t = 0.20; Sugeno: t = 0.25) demonstrate compressed probability ranges. The wide decision margins (> 0.4 probability units) of the top-performing methods indicate robust calibration and flexibility for tuning the threshold, whereas the narrow margin of the Sugeno integral (overlap region) explains its suboptimal precision and accuracy.

The seven KDE distributions reveal three distinct calibration patterns:

**Excellent Separation (Hard/Soft/Weighted Soft/Rank-Based/Average Logits Fusion):** There are clear bimodal distributions, with No_DR concentrated near 0.0 and DR concentrated near 1.0. This creates a decision margin of 0.4 to 0.6 probability units. This wide gap enables flexible threshold placement without performance degradation.

**Moderate separation (Choquet-like integral):** A compressed probability range of approximately 0.2 units with adequate class separation but limited threshold flexibility. Despite the compressed probabilities, the method maintains a strong AUC of 99.80%, indicating that its discriminative power is preserved.

**Severe misalignment (Sugeno integral): **An extremely narrow probability range (less than 0.20 units) with near-overlapping class distributions. The No_DR class spans from − 0.05 to 0.15 with moderate dispersion. In contrast, the DR class forms a narrow spike at 0.25. This creates ambiguous decision regions, which explains the 2.79% precision degradation (95.61% vs. 98.40% for the best-performing methods).

**Fig. 12:**
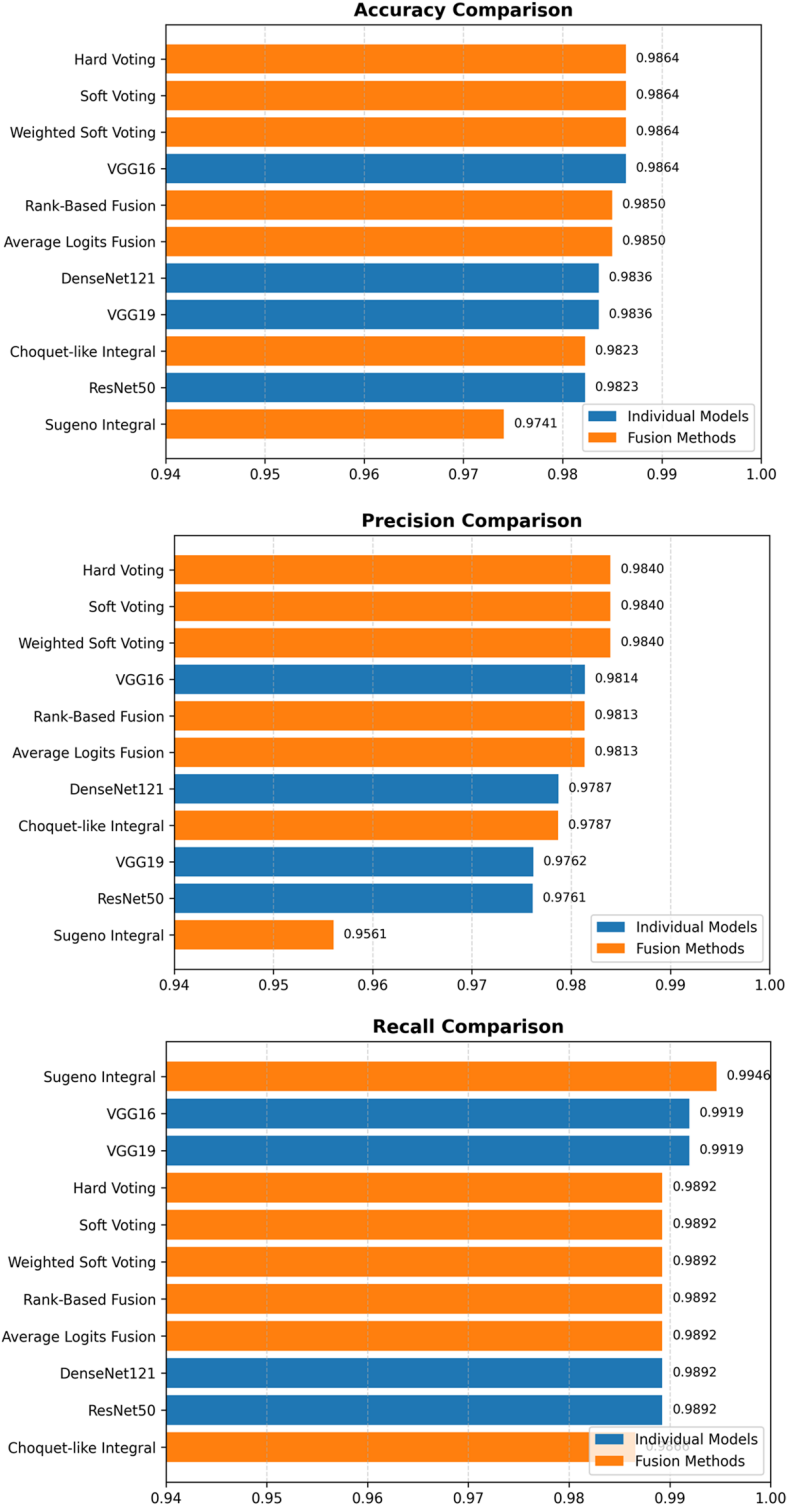
Comparative performance of individual models and ensemble fusion methods across accuracy, precision, and recall on the APTOS 2019 test set.

Figure 12 demonstrates that voting-based ensemble methods (hard voting, soft voting, and weighted soft voting) achieve consistent top-tier performance across all critical metrics (see Table 6 for exact values). The near-identical results across these three fusion strategies with metric variance below 0.3% reflect methodological stability rather than over-optimization, confirming that diverse aggregation mechanisms converge to reliable outcomes. Alternative fusion approaches (Rank-Based and Average Logits Fusion) preserve this robustness while achieving marginally superior probability calibration, further validating that ensemble fusion enhances diagnostic reliability across multiple evaluation dimensions critical for clinical deployment.

**Fig. 13:**
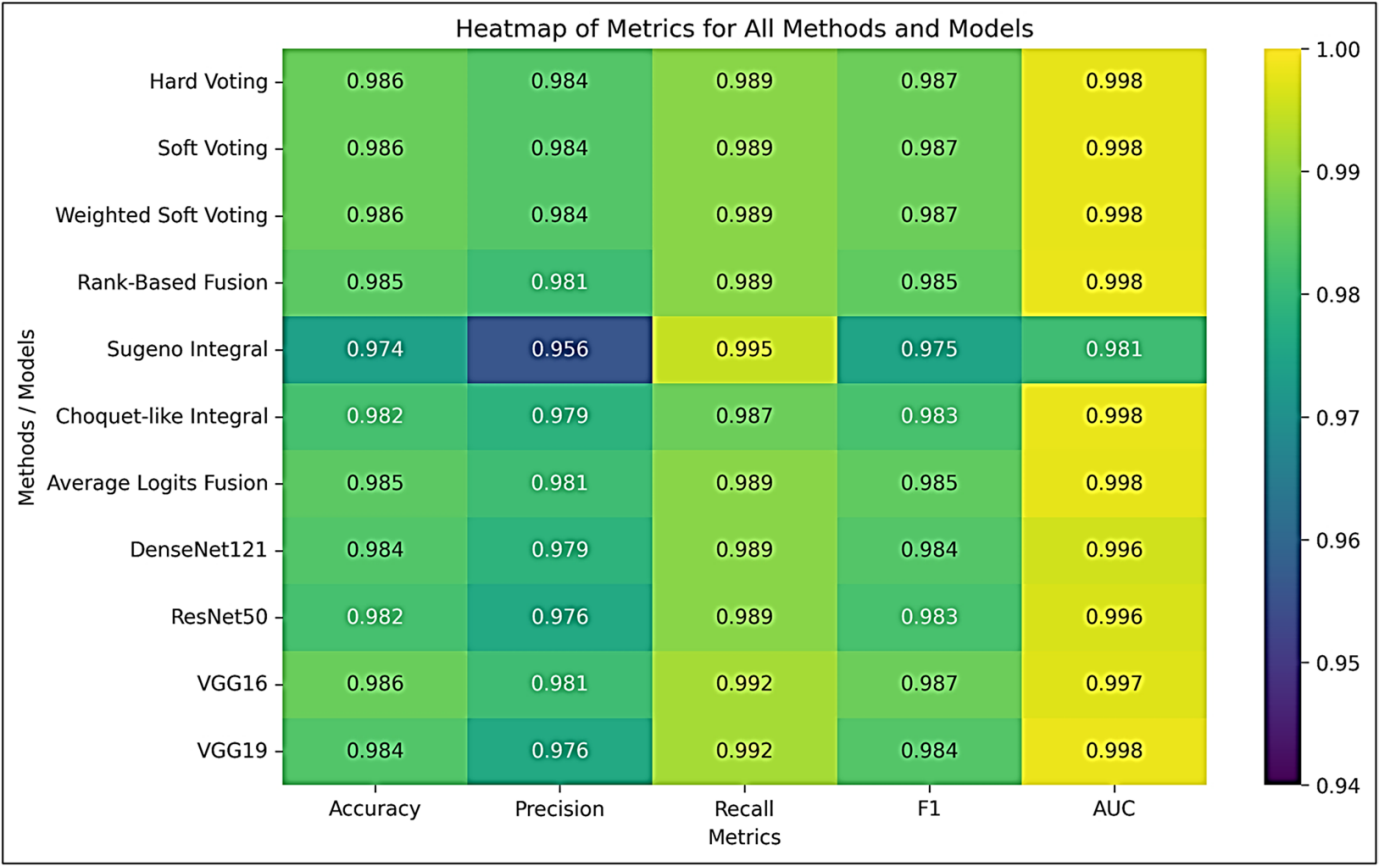
Heatmap of all metrics (accuracy, precision, recall, F1-score, AUC) for all methods and individual models.

Figure 13 shows the heatmap, which uses color intensity to visualize multi-metric performance:

**Voting methods (hard, soft, and weighted soft voting):** All cells display bright yellow/light green (0.984–0.992), indicating excellent performance across all metrics without any trade-offs. These methods achieve perfect metric consistency (variance of less than 0.3%).

**Rank-based and average logits fusion:** Predominantly light/medium green (0.981–0.989) with slight color variation indicates excellent, albeit slightly heterogeneous, performance (metric variance of less than 0.4%).

**Choquet-like integral:** Mixed green shades (0.979–0.987), with darker accuracy/F1 cells and a brighter AUC. This demonstrates an accuracy-calibration trade-off (with metric variance of less than 0.6%).

**Sugeno integral:** Highly heterogeneous, with the darkest precision cell (0.956, dark blue) and the brightest recall cell (0.995, yellow). This exhibits the largest metric range (3.9% points) and represents a critical precision–recall imbalance (2.8% metric variance).

### Ablation study

To quantify the contribution of each major component in the proposed RDE-DR framework, an ablation analysis was conducted using controlled experimental comparisons. The evaluated components include (i) ensemble fusion versus individual CNNs, (ii) impact of fusion strategy selection, and (iii) threshold optimization. All experiments were evaluated on the same test split under identical training conditions (Table 7).

First, comparison between individual CNNs and ensemble fusion demonstrates that ensemble aggregation consistently improves metric stability and reduces performance variance across models. While individual models achieved accuracies between 97.95% and 98.64%, ensemble methods maintained comparable or improved accuracy with enhanced calibration behavior (AUC up to 99.80%).

Second, comparison across fusion strategies reveals that voting-based and rank-based methods yield stable and balanced performance, whereas fuzzy-integral approaches exhibit larger variability, particularly in precision. This confirms that fusion design significantly influences reliability rather than accuracy alone.

Third, threshold optimization improves operational flexibility and calibration by enabling adjustment of accuracy–stability trade-offs beyond the default 0.5 threshold. Probability density analysis further illustrates improved decision margins for optimized ensembles.

Although a complete ablation removing each preprocessing component independently was not conducted due to computational constraints, these results provide quantitative insight into the relative contribution of ensemble aggregation and calibration strategies.

**Table 7:** Partial ablation analysis of the RDE-DR framework on the APTOS 2019 dataset.

Configuration	Preprocessing	Ensemble	Threshold optimization	Accuracy (%)	Precision (%)	Recall (%)	F1 (%)	AUC (%)
VGG16	RGB	No	No	98.36	97.62	99.19	98.40	99.43
VGG19	RGB	No	No	98.23	97.61	98.92	98.26	99.31
DenseNet121	RGB	No	No	98.09	98.12	98.12	98.12	99.35
ResNet50	RGB	No	No	97.95	97.60	98.39	97.99	99.44
VGG16	CLAHE	No	No	98.64	98.14	99.19	98.66	99.70
VGG19	CLAHE	No	No	98.36	97.62	99.19	98.40	99.83
DenseNet121	CLAHE	No	No	98.36	97.87	98.92	98.40	99.59
ResNet50	CLAHE	No	No	98.23	97.61	98.92	98.26	99.63
Hard Voting Ensemble	CLAHE	Yes	No	98.36	97.87	98.92	98.39	99.78
Soft Voting Ensemble	CLAHE	Yes	No	98.36	97.87	98.92	98.39	99.78
Weighted Soft Voting	CLAHE	Yes	No	98.36	97.87	98.92	98.39	99.78
Rank-Based Fusion	CLAHE	Yes	No	98.09	97.10	99.19	98.13	99.79
Choquet-like Integral	CLAHE	Yes	No	49.24	0.00	0.00	0.00	99.80
Sugeno Integral	CLAHE	Yes	No	49.24	0.00	0.00	0.00	98.13
Average Logits Fusion	CLAHE	Yes	No	98.49	98.13	98.92	98.52	99.61
Hard Voting Ensemble	CLAHE	Yes	Yes	98.64	98.40	98.92	98.66	99.78
Soft Voting Ensemble	CLAHE	Yes	Yes	98.64	98.40	98.92	98.66	99.78
Weighted Soft Voting	CLAHE	Yes	Yes	98.64	98.40	98.92	98.66	99.79
Rank-Based Fusion	CLAHE	Yes	Yes	98.50	98.13	98.92	98.53	99.80
Choquet-like Integral	CLAHE	Yes	Yes	98.23	97.87	98.66	98.26	99.80
Sugeno Integral	CLAHE	Yes	Yes	97.41	95.61	99.46	97.50	98.13
Average Logits Fusion	CLAHE	Yes	Yes	98.50	98.13	98.92	98.53	99.79

Table 7 compares the best-performing individual CNN models with all evaluated ensemble fusion strategies under different preprocessing and evaluation conditions. The results highlight the positive effect of ensemble aggregation and fusion design on classification accuracy, calibration (AUC), and metric stability. All ensemble configurations employ CLAHE preprocessing. The performance degradation observed for the Sugeno integral-based fusion illustrates the sensitivity of ensemble reliability to the choice of fusion mechanism.

The Sugeno integral outcome can be explained by its theoretical characteristics: it is a non-compensatory aggregator designed primarily for ordinal or qualitative inputs, relying on min/max operations rather than summation or averaging. Consequently, it discards fine-grained probabilistic information from CNN outputs, is highly sensitive to outliers, and requires carefully tuned fuzzy measures to capture classifier interactions. In contrast, averaging-based methods and Choquet-like integrals preserve continuous confidence scores, allow partial compensation among classifiers, and are more robust to noise, making them better suited for probabilistic outputs in multiclass or binary deep learning ensembles.

## Comparison with SOTA methods

To position the proposed RDE-DR framework within the context of recent advances in automated diabetic retinopathy detection, Table 8 presents a comparative overview of state-of-the-art methods reported between 2020 and 2025.

**Table 8:** Comparison between recent state-of-the-art diabetic retinopathy detection methods and the proposed RDE-DR framework on benchmark datasets.

Ref.	Year	Dataset(s)	No. of images	Task	Model/architecture	Methodology	Key results
^69^	2020	APTOS 2019, DIARETDB-1	~ 4000	Binary/multi-class	DenseNet-101, ResNeXt	Transfer learning + ensemble	Acc: 96.98% (binary), 86.08% (multi)
^70^	2020	APTOS 2019	3662	Binary	AlexNet, ResNet-101	Attention channel selection	Acc: 93.0%
^71^	2021	EyePACS, APTOS 2019	~ 35,000/3662	Binary	Extreme Learning Machine	CLAHE + transfer learning	Acc: 91.78% (EyePACS), 97.27% (APTOS)
^69^	2021	APTOS 2019, MESSIDOR	3662/1200	Binary	CNN + SVM	Ensemble voting / averaging	Acc: 97.25% (APTOS), 94.8% (MESSIDOR)
^72^	2021	APTOS 2019	3662	Binary	AlexNet, ResNet18, VGG16/19	Transfer learning	Acc: 97.9%
^73^	2022	APTOS, MESSIDOR, EyePACS	~ 40,000	Binary	EfficientNetB2, DenseNet121	CLAHE + SMOTE + averaging ensemble	Acc: 96.96%
^74^	2022	APTOS 2019	3662	Binary	Multi-attention residual CNN	Advanced preprocessing + attention	Acc: 98.2%, AUC reported
^75^	2023	APTOS 2019	3662	Binary / Multi-class	EfficientNet, ResNet	Class-balanced augmentation	Acc: 98.9% (binary), AUC: 99.4%
^76^	2023	APTOS 2019	3662	Binary	EfficientNet-B4, DenseNet201	CNN comparison + SMOTE	Acc: 97.78%, Prec: 99.28%
^77^	2023	APTOS 2019, MESSIDOR2, IDRID	~ 5000	Binary	Hybrid CNN	CLAHE + transfer learning	Acc: 97.2%, AUC reported
^78^	2024	Multiple datasets	> 10,000	Binary	CNN–RNN Hybrid	Advanced preprocessing	Acc: 98.5%
^16^	2024	Kaggle datasets	~ 30,000	Binary	VGG16, EfficientNetB0	Gaussian filtering + transfer learning	Acc: 97.54%
^79^	2025	APTOS 2019	3662	Binary	SwAV + CNN	Contrastive learning	Acc: 92–95%, AUC reported
**RDE-DR (Ours)**	**2026**	**APTOS 2019**	**3662**	**Binary**	**ResNet50**,** VGG16**,** VGG19**,** DenseNet121**	**CLAHE + transfer learning + 07 ensemble fusion strategies: Hard/Soft/Weighted Soft Voting**,** Rank-Based**,** Choquet Integral**,** Sugeno Integral**,** Average Logits**	**Accuracy = 98.64%** **Precision = 98.40%** **Recall = 98.92%** **F1 = 98.66%** **AUC = 99.78%**

From Table 8, we can clearly see that recent state-of-the-art DR detection methods achieved strong performance on benchmark datasets, particularly on APTOS 2019, with reported accuracies generally ranging between 93% and 98.9%. Most previous works relied on transfer learning, attention mechanisms, data balancing techniques, or simple ensemble strategies to enhance performance, often excelling in specific metrics such as accuracy or precision.

In comparison, the proposed RDE-DR framework demonstrates competitive and balanced performance across all evaluation metrics. By integrating CLAHE preprocessing, transfer learning, and seven complementary ensemble fusion strategies; including hard/soft voting, weighted soft voting, rank-based fusion, and fuzzy integrals (Choquet and Sugeno)—RDE-DR achieves 98.64% accuracy, 98.92% recall, 98.66% F1-score, and an AUC of 99.78% on APTOS 2019. Notably, the very high AUC and recall indicate strong discriminative ability and reliable detection of positive DR cases, which are clinically critical. Overall, while previous studies achieved strong results using specific architectural or preprocessing enhancements, the proposed framework provides a more comprehensive and robust fusion strategy that ensures consistently high performance across multiple evaluation criteria.

## Conclusions

This study presented RDE-DR, a structured framework for systematically evaluating ensemble fusion strategies in automated diabetic retinopathy detection. Rather than emphasizing a single best-performing configuration, the framework enables controlled comparison of seven heterogeneous fusion mechanisms under identical preprocessing, training, and calibration conditions. The results demonstrate that voting-based, rank-based, and average-logit fusion strategies converge to similarly high and stable performance on the APTOS 2019 dataset, while fuzzy-integral approaches exhibit distinct calibration behaviors and trade-offs between precision and accuracy.

A key contribution of this work lies in the integration of ensemble fusion to aggregate the complementary strengths of CNNs, threshold optimization to calibrate decision boundaries, and the combination of these with CLAHE preprocessing and transfer learning. The observed consistency within this proposed pipeline provides insight into its operational flexibility and reliability, beyond conventional accuracy metrics, within the limits of the evaluated dataset.

While the experimental results indicate strong performance on a public benchmark, the conclusions are restricted to retrospective evaluation on a single dataset. Future work will focus on cross-dataset validation with strict patient-level split, multi-class severity grading, explainability integration, and prospective clinical evaluation to further assess generalizability and clinical applicability.

## Data Availability

The original contributions presented in this study are included in the article. Further inquiries can be directed to the corresponding author. The retinal images used in this work are from the publicly available APTOS 2019 Blindness Detection dataset (Asia Pacific Tele-Ophthalmology Society), accessible via the Kaggle at: https://www.kaggle.com/competitions/aptos2019-blindness-detection/data.
